# Common autoimmune diseases and urticaria: the causal relationship from a bidirectional two-sample mendelian randomization study

**DOI:** 10.3389/fimmu.2023.1280135

**Published:** 2023-11-02

**Authors:** Mingyi Yang, Yani Su, Ke Xu, Pengfei Wen, Binfei Zhang, Jianbin Guo, Kai Nan, Peng Yang, Xiaolong Shao, Lin Liu, Zhi Yang, Peng Xu

**Affiliations:** Department of Joint Surgery, HongHui Hospital, Xi’an Jiaotong University, Xi’an, Shaanxi, China

**Keywords:** urticaria, rheumatoid arthritis, systemic lupus erythematosus, genetic, causal

## Abstract

**Objective:**

The immune response assumes a pivotal role in the underlying mechanisms of urticaria pathogenesis. The present study delves into an investigation of the genetic causal connections between urticaria and prevalent autoimmune afflictions, notably rheumatoid arthritis (RA), systemic lupus erythematosus (SLE), ulcerative colitis (UC), and Crohn’s disease (CD).

**Methods:**

A bidirectional two-sample Mendelian randomization (MR) analysis was conducted to investigate the causal relationships involving four autoimmune diseases and urticaria. The genome-wide association study (GWAS) summary data of four autoimmune disease were sourced from the IEU OpenGWAS database. The GWAS summary data for urticaria were derived from the Finnish consortium dataset. The principal analytical approach employed in this study was the random-effects inverse variance weighted (IVW) method. Subsequently, a series of sensitivity analyses were performed, encompassing assessments of heterogeneity, horizontal pleiotropy, outliers, “Leave-one-out” analyses, and tests for adherence to the assumption of normal distribution.

**Results:**

The random-effects IVW analysis indicate a positive genetic causal association between RA and urticaria (P < 0.001, OR 95% CI = 1.091 [1.051-1.133]). Conversely, SLE, UC, and CD do not exhibit a significant genetic causal relationship with urticaria. The reverse MR analysis reveals a positive genetic causal linkage between urticaria and SLE (P = 0.026, OR 95% CI = 1.289 [1.031-1.612]). However, the analysis demonstrates no substantial genetic causal relationship between urticaria and RA, UC, or CD. Importantly, the genetic causal assessment absence of heterogeneity, horizontal pleiotropy, and outliers. Furthermore, it remains unaffected by any individual single nucleotide polymorphism (SNP), demonstrating adherence to a normal distribution.

**Conclusion:**

This investigation establishing RA as a predisposing factor for urticaria. Moreover, urticaria as a plausible risk determinant for SLE. Heightened vigilance is recommended among RA patients to monitor the manifestation of urticaria within clinical settings. Similarly, individuals afflicted by urticaria should duly acknowledge the prospective susceptibility to SLE.

## 1 Introduction

Urticaria, commonly referred to as “hives,” possesses an extensive and illustrious historical trajectory within the annals of documented medical history, tracing its origins back to no later than the 10th century B.C., when it was denoted as ‘Feng Yin Zheng’ in the medical texts of ancient China (1). Urticaria stands as a prevalent and multifaceted inflammatory dermatological ailment, exhibiting a global lifetime prevalence that extends to 20%. This condition arises from the activation and degranulation of cutaneous mast cells, subsequently precipitating the liberation of histamine and other assorted mediators. These biochemical cascades give rise to the activation of sensory nerve pathways, vasodilation, plasmatic exosmosis, and cellular recruitment (2). Consequent stimulation of sensory nerve fibers leads to pruritus and reflex erythema, while angioedema emanates from the concurrent vasodilatory response, plasmatic extravasation, and cellular influx. Consequently, the clinical manifestation of urticaria predominantly takes the form of a rash, angioedema, or a combination thereof (3). The etiological underpinnings of urticaria encompass localized vasodilation, heightened capillary permeability, and plasmatic effusion (4). Notably, its paramount characteristic manifests as an acute or chronic pruritic eruption, tending to elicit friction-induced exacerbation rather than overt scratching. Urticarial eruptions may manifest universally across the skin landscape, typically distributed locally or diffusely throughout the corporeal expanse. The affliction of itchiness proves insufferable, with resultant eruptions escalating post-scratching, exhibiting a predilection for nocturnal exacerbation, and enduring for extended periods prior to subsiding (4). Concomitant systemic symptoms, such as pyrexia, fatigue, respiratory or gastrointestinal perturbations, and arthralgia (5). Notably, there has been a perceptible exponential surge in the incidence of urticaria over recent years (6). Approximating 1% of the global populace, primarily encompassing young and middle-aged women, falls victim to the scourge of urticarial affliction (7). Furthermore, substantiating evidence underscores the potential of urticaria to instigate anxiety, depression, sleep disturbances, and sexual dysfunction, thereby exerting a profound detraction from quality of life and engendering considerable sociocultural detriment (8, 9).

Histamine, released through mast cell degranulation, initiates vasodilation and vascular permeability, culminating in cutaneous edema of urticaria. Beyond histamine, an immediate release of various factors such as tumor necrosis factor-α (TNF-α), serotonin, proteases, and proteoglycans also occur, manifesting direct or indirect implications in the pathogenesis of urticaria. Furthermore, subsequent to mast cell activation, an array of molecules like prostaglandins, leukotrienes, cytokines, and chemokines are generated at disparate intervals, collectively contributing to the diverse clinical presentations and evolutionary trajectory of the disorder (10). The immune response significantly underpins the pathophysiological intricacies of urticaria. While the classical immunoglobulin (Ig) E-mediated type I hypersensitivity reaction has historically been paramount in understanding mast cell activation, recent investigations challenge the notion that interactions between allergen-bound IgE and mast cells account for the majority of urticarial cases (10). Instead, prevailing evidence underscores the prominence of an alternate immune mechanism: type II hypersensitivity reactions facilitated by IgG autoantibodies binding to IgE receptors on mast cells, superseding type I hypersensitivity. Moreover, type III hypersensitivity reactions, through engagement with circulating immune complexes and mast cells equipped with IgG and IgM Fc receptors, along with infrequent instances of type IV hypersensitivity, mediated by T cell interactions, contribute to mast cell activation and histamine release (10). In light of these intricate immune pathways, the compelling role of the immune response in shaping urticarial pathogenesis necessitates heightened consideration of the interplay between urticaria and autoimmune maladies.

Urticaria is typically categorized into two primary forms: inducible urticaria and spontaneous urticaria. Spontaneous urticaria further encompasses acute spontaneous urticaria (ASU), defined as lasting less than six weeks, and chronic spontaneous urticaria (CSU), characterized by a duration exceeding six weeks (3). Considerable circumstantial evidence supports the notion that CSU possesses autoimmune characteristics, with an estimated 35-40% of CSU cases being attributed to autoimmune mechanisms. CSU represents a debilitating condition primarily driven by mast cell activity, featuring recurrent episodes of urticaria and/or angioedema. CSU, as per the established criteria, is demarcated by the presence of unprovoked and transient rash and pruritus persisting for a duration exceeding six weeks (11). Within the spectrum of CSU, Type I autoimmune (autoallergic) CSU is notably linked to the presence of IgE antibodies directed against autoantigens, such as thyroid peroxidase and IL-24. Conversely, Type IIb autoimmune CSU is characterized by the involvement of autoantibodies that activate mast cells, typically through mechanisms involving IgE and FC-ϵ RI. The identification of Type IIb autoimmune CSU necessitates strict diagnostic criteria, encompassing triple positive autoserum skin tests, IgG autoantibody immunoassays, and basophil activation tests. It is worth noting, however, that this autoimmune subtype is discernable in less than 10% of individuals diagnosed with CSU. Type IIb autoimmune CSU is further typified by its distinct clinical features, including heightened disease severity, concurrent autoimmune comorbidities, diminished total IgE levels, elevated IgG-anti-thyroid peroxidase levels, and specific hematological aberrations like alkaline granulocytopenia and acidocytopenia. This subtype is also marked by suboptimal responsiveness to conventional antihistamines and omalizumab, but exhibits a favorable response to cyclosporine (11, 12). Furthermore, there is compelling evidence indicating that CSU not only manifests autoimmune attributes but may also co-occur with other autoimmune disorders (13). Autoimmunity is thus considered a prevailing factor in CSU etiology, frequently accompanied by the presence of multiple autoimmune conditions. Numerous independent studies have explored the prevalence of CSU and CSU-like dermatological manifestations in systemic lupus erythematosus (SLE) patients, revealing comorbidities that range from 0% to 21.9% in adult individuals and 0.4% to 27.5% for the latter condition (14). In pediatric SLE cases, CSU incidence varies from 0% to 1.2%, with CSU-like rashes appearing at rates ranging from 4.5% to 12% (14). Notably, the majority of studies report comorbidities between CSU and rheumatoid arthritis (RA) exceeding 1% (15). This cutaneous manifestation is frequently observed in autoimmune diseases such as SLE and RA (13). A comprehensive follow-up study involving nearly 13,000 patients diagnosed with chronic urticaria unveiled a notable prevalence of RA and SLE. Furthermore, a high incidence of positive rheumatoid factors and antinuclear antibodies was observed among patients afflicted with chronic urticaria (16). A separate investigation encompassing 9,332 patients with chronic urticaria established a robust association between chronic urticaria and SLE, as well as inflammatory bowel disease (IBD) (17). IBD primarily comprises ulcerative colitis (UC) and Crohn’s disease (CD). Many prior research endeavors concerning the link between urticaria and autoimmune disorders have concentrated on RA, SLE, and IBD. Notably, RA, SLE, and IBD are prevalent autoimmune disorders within clinical practice. Consequently, exploring their association with urticaria bears substantial clinical significance, underscoring the need for further investigations into the intricate relationship between urticaria and these common autoimmune conditions, including RA, SLE, and IBD.

Mendelian Randomization (MR) analyses investigate the causal ramifications of exposures on outcomes by leveraging genetic variation as instrumental variables (IVs). Predominantly, single nucleotide polymorphisms (SNPs) constitute the prevailing genetic variants of choice. The underlying tenet derives from Mendel’s second law of inheritance, whereby alleles are fortuitously allocated at conception. This method parallels the conventional randomized controlled trial (RCT), wherein patients are haphazardly allocated to both treatment and control cohorts. Exploiting the fortuitous allocation of genetic variance during gametic formation, largely impervious to environmental and lifestyle influences, MR demonstrates its capacity to mitigate the confounding effects and reverse causal associations (18). The causal nexus between urticaria and gut microbiota has been subjected to scrutiny via MR analysis (19). Urticaria is a clinically diverse condition comprising various subtypes characterized by distinct clinical manifestations, triggering factors, and underlying pathophysiological mechanisms. Regrettably, comprehensive genome-wide association study (GWAS) datasets specific to individual urticaria subtypes remain conspicuously absent. Consequently, this study employs GWAS data encompassing the entire urticaria for the purposes of analysis. In this inquiry, we have employed a bidirectional two-sample MR approach to meticulously probe the genetic underpinnings of causation between urticaria and prevalent autoimmune conditions, encompassing RA, SLE, UC, and CD.

## 2 Materials and methods

### 2.1 Study design

A bidirectional two-sample MR analysis was performed for autoimmune diseases (RA, SLE, UC, CD) and urticaria. The analytical process involved two primary steps. Initially, an MR analysis was conducted employing RA, SLE, UC, and CD as exposures, and urticaria as the outcome. Subsequently, a reverse MR analysis was performed, employing urticaria as the exposure, and RA, SLE, UC, and CD as the outcomes. It is noteworthy that this investigation rigorously adheres to the foundational assumptions underpinning MR analysis, encompassing: 1) the establishment of a robust association between the IVs and the exposure, 2) independence of IVs from potential confounding factors, and 3) the direct influence of IVs on the outcome exclusively via the exposure.

### 2.2 Data source

The GWAS summary data of RA, SLE, UC, and CD were sourced from the IEU OpenGWAS database, accessible at https://gwas.mrcieu.ac.uk/. Notably, the GWAS summary data for RA encompasses a sample size of 58,284 individuals, involving 13,108,512 SNPs. Likewise, the SLE GWAS summary data comprises 14,267 samples with 7,071,163 SNPs, the UC dataset involves 26,405 samples and 1,116,795 SNPs, while the CD dataset encompasses 20,883 samples and 12,276,506 SNPs. The GWAS summary data concerning urticaria emanates from the Finnish consortium, as found at https://www.finngen.fi/. This dataset encompasses a considerable sample size of 217,530 individuals includes males and females, and features 16,380,466 SNPs. All case subjects were ascertained through the application of the M13 code in the International Classification of Diseases-Tenth Revision (ICD-10). The genotyping procedures were executed using illumina (Illumina Inc, San Diego) and Affymetrix chip arrays (Thermo Fisher Scientific, Santa Clara, CA, USA). For further insights into the data employed, interested parties are encouraged to access the FinnGen website. It is crucial to emphasize that all participants encompassed in both the autoimmune disease and urticaria cohorts shared a common European ancestry. Furthermore, the data harnessed for this investigation is sourced exclusively from publicly available databases. As such, the study is exempt from necessitating an ethical statement and informed consent due to the data’s publicly accessible nature. **Supplementary Table 1** offers comprehensive details pertaining to the data implemented in this analysis.

### 2.3 IVs selection

It is well known that the rigorous selection of IVs constitutes the foundation for ensuring the robustness of MR analysis outcomes. Initially, IVs exhibit a robust association with the exposure, with the criteria for a substantial correlation established as follows: P < 5 x 10^-8^ and F statistic > 10. The computation of the F statistic is carried out employing the formula: F = R^2^(N-K-1)/K(1-R^2^). Subsequently, a meticulous screening process is executed to exclude the influence of linkage disequilibrium (LD), whereby solely SNPs exhibiting minimal LD (LD r^2^ < 0.001 and a clump distance > 10,000 kb) are retained as IVs. In addition, the chosen IVs must exhibit no discernible correlation with the outcome, thus necessitating the removal of SNPs associated with the outcome, predicated on a correlation threshold of: P < 5 x 10^-8^. To further mitigate confounding variables, the PhenoScanner database is utilized, serving to mitigate the impact of extraneous factors. The spectrum of confounding variables considered within this investigation encompasses but is not restricted to various elements. In the context of urticaria, these encompass factors such as obesity, advanced age, vitamin D deficiency, and infection (20, 21). Similarly, for RA, confounding factors encompass, though are not limited to, variables such as smoking, obesity, and gender (22). For SLE, confounding variables span dimensions including, but not confined to, smoking, alcohol consumption, obesity, and vitamin D deficiency (23). The variables influencing UC include factors such as family history of inflammatory bowel disease, past gastrointestinal infections, and oral contraceptive use (24). Lastly, in the context of CD, variables including, but not restricted to, smoking, family history of inflammatory bowel disease, prior gastrointestinal infections, and oral contraceptive usage, are accounted for as potential confounders (25). Ultimately, SNPs characterized by palindromic sequences and intermediate allele frequencies are systematically excluded from the analytical process.

### 2.4 MR analysis

The primary analytical approach employed in this study is the random-effects inverse variance weighted (IVW) method. We incorporated four supplementary methodologies: MR Egger, weighted median, simple mode, and weighted mode. Furthermore, for validation results, we engaged in the utilization of the maximum likelihood, penalized weighted median, and fixed-effects IVW techniques. Notably, within this array of methods, the random-effects IVW approach exhibited notable power in terms of its statistical properties (26), consequently prompting a heightened reliance on its analytical outcomes. The IVW method operates by leveraging meta-analytic principles to amalgamate Wald ratio estimates pertaining to the causal impact deduced from distinct SNPs. This amalgamation furnishes a coherent appraisal of the causal effect originating from the exposure on the outcome, contingent upon the fulfillment of IVs assumptions for each genetic variant (27). In the absence of horizontal pleiotropy, the IVW methodology demonstrates an aptitude for generating relatively consistent and accurate causal evaluations. This is achieved by effectuating a meta-analytical strategy to amalgamate Wald estimates associated with each IV (28).

### 2.5 Sensitivity analysis

Sensitivity analysis stands as a pivotal methodology employed to evaluate the robustness of results derived from MR analyses. Given the inherent diversity within GWAS summary data, the potential existence of heterogeneity in MR analyses has traditionally been not dismissed as a factor influencing the veracity of outcomes in genetic causal inference. Our investigation integrates two distinct approaches to scrutinize the presence of heterogeneity: Cochran’s Q statistic applied to MR-IVW and Rucker’s Q statistic employed for MR Egger. It is imperative to underscore that the absence of horizontal pleiotropy constitutes a fundamental prerequisite for the validity of outcomes in MR analyses. To assess the potential presence of horizontal pleiotropy, the intercept test within the MR Egger framework is employed. Additionally, the MR pleiotropy residual sum and outlier (MR-PRESSO) methodology is harnessed due to its heightened statistical power in detecting horizontal pleiotropic effects (29). The MR-PRESSO technique is adeptly applied to identify and subsequently exclude outliers during the MR analysis. Furthermore, a “Leave-one-out” analysis is undertaken to ascertain the potential impact of individual SNPs on the genetic causal inference. Lastly, the MR robust adjusted profile score (MR-RAPS) method is invoked to subject the distribution of MR analysis outcomes to a test of normality.

### 2.6 Statistical analysis

The “TwoSampleMR” software package was employed to conduct two-sample MR analyses, while the “MRPRESSO” software package was employed to execute the MR-PRESSO test. All statistical analyses were carried out utilizing R version 4.1.2. A significance level of P < 0.05 was adopted to infer genetic causation. Specifically, when the P-value < 0.05 and Odds Ratio (OR) > 1 ​was indicative of a positive genetic causal relationship, whereas an OR < 1 implied the presence of a negative genetic causal association. Moreover, instances where the P-value > 0.05 indicated the absence of heterogeneity, horizontal pleiotropy, and adhering to the assumptions of a normal distribution.

## 3 Results

### 3.1 Genetic causality between exposures (RA, SLE, UC, CD) and outcome (urticaria)

**Figure 1:**
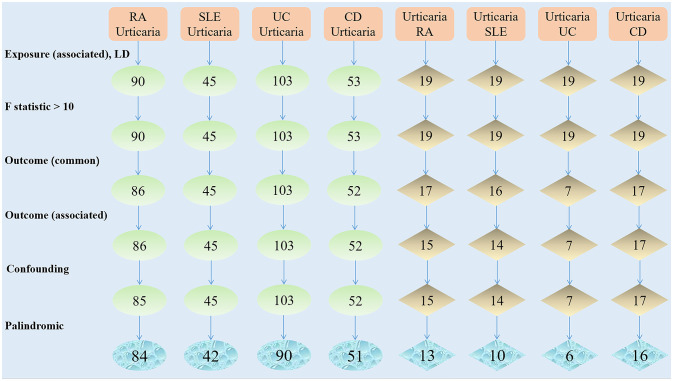
The selection process of instrumental variable.

We identified a total of 90 SNPs exhibiting significant associations with RA. The 86 SNPs was gleaned from GWAS summary data for urticaria. None of the identified SNPs displayed any discernible correlation with urticaria. One confounder SNP (rs1883832) and one palindromic SNP (rs34536443) were excluded from further consideration. Subsequently, the remaining set of 84 SNPs were designated as IVs, as detailed in **Supplementary Table 2**. Moreover, in the context of SLE, we procured a contingent of 45 SNPs that demonstrated robust associations. A corresponding set of 45 SNPs was extracted from the urticaria GWAS summary data. No SNPs exhibited any significant link with urticaria or potential confounding factors. Three palindromic SNPs (rs2736332, rs28834423, rs28361029) were excluded. Consequently, the residual 42 SNPs were designated as IVs and are cataloged in **Supplementary Table 3**. Furthermore, our investigation into UC yielded 103 SNPs that exhibited robust statistical associations. Concomitantly, a collection of 103 SNPs was extracted from the urticaria GWAS summary data. Notably, none of the identified SNPs demonstrated any significant correlation with urticaria or any associated confounding variables. In pursuit of methodological rigor, a subset of 13 palindromic SNPs (rs12565572, rs1543247, rs16905158, rs17066875, rs17103762, rs17141433, rs17152619, rs2249237, rs271461, rs6008068, rs6029933, rs6114452, rs9377315) was excluded from further consideration. The remaining cohort of 90 SNPs was subsequently endorsed as IVs and is presented in **Supplementary Table 4**. Subsequently, in the context of CD, 53 SNPs was discerned, showcasing robust associations. Correspondingly, 52 SNPs were obtained from the urticaria GWAS summary data. No SNPs exhibited any appreciable correlation with urticaria or potential confounding factors. One palindromic SNP (rs12692254) was excluded from further analysis. Consequently, the remaining 51 SNPs were validated as IVs and are tabulated in **Supplementary Table 5**. The comprehensive procedure undertaken for the selection of instrumental variables is visually delineated in **Figure 1**.

**Figure 2:**
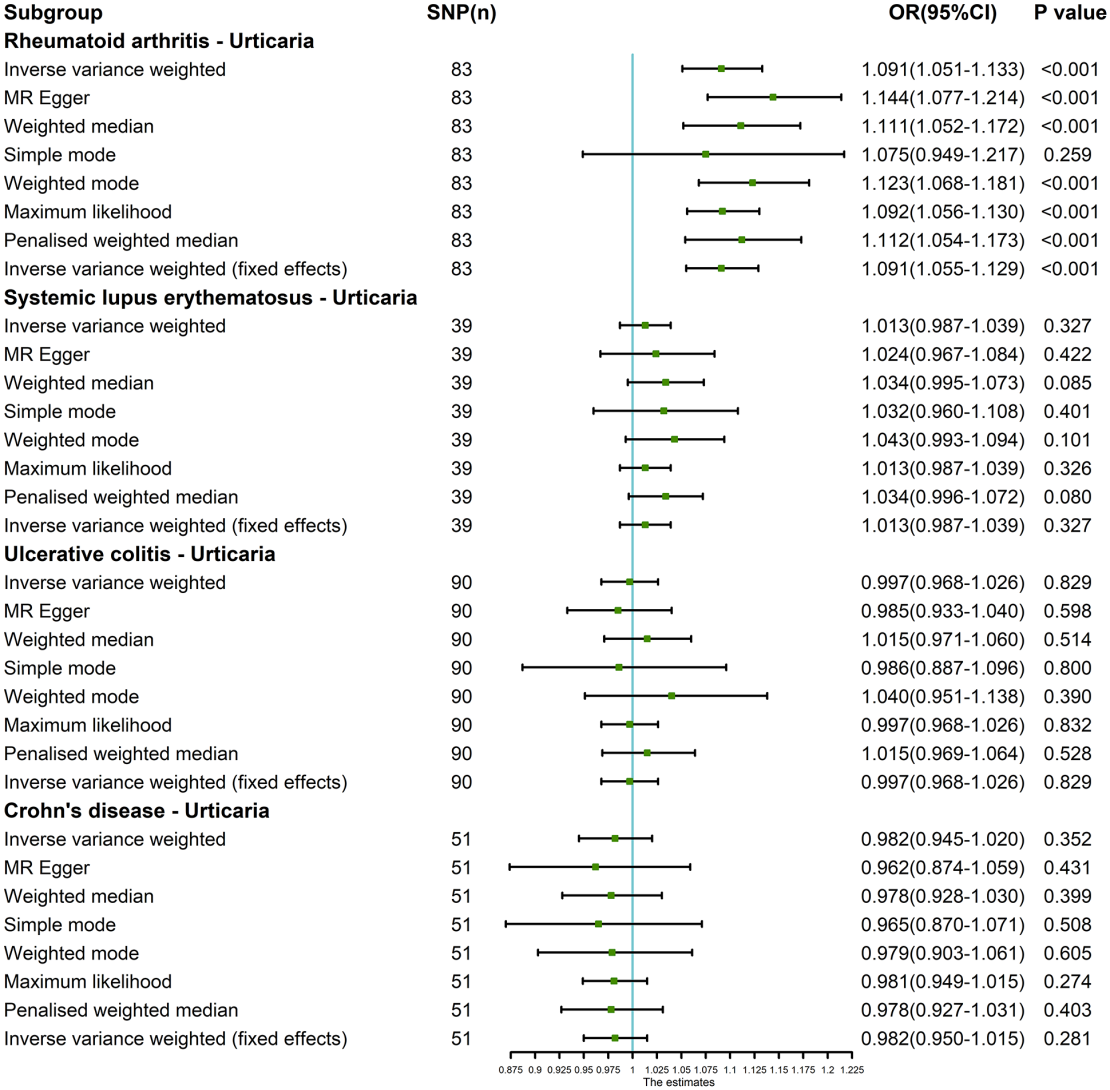
The MR analysis results of exposures (RA, SLE, UC and CD) and outcome (urticaria). The analysis employed eight methods, namely random-effects IVW, MR Egger, weighted median, simple mode, weighted mode, maximum likelihood, penalized weighted median, and fixed-effects IVW.

**Figure 3:**
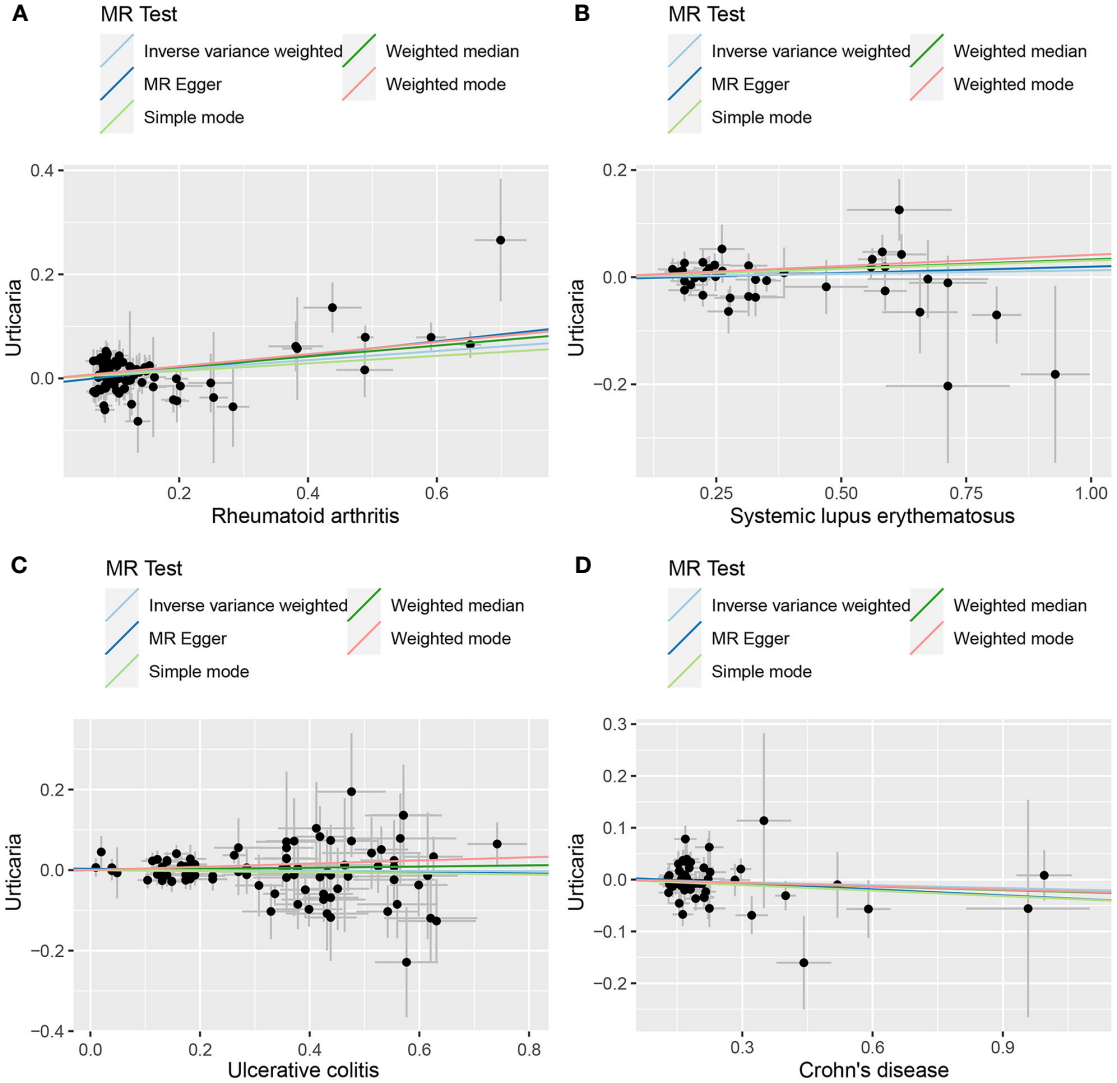
The scatter plot of MR analysis between exposures (RA, SLE, UC and CD) and outcome (urticaria). **(A)** RA and urticaria; **(B)** SLE and urticaria; **(C)** UC and urticaria; **(D)** CD and urticaria.

The outcomes of the random-effects IVW analysis revealed a positive genetic causal relationship between RA and urticaria (P < 0.001, odds ratio [OR] 95% confidence interval [CI] = 1.067 [1.028 - 1.107]). Assessments of heterogeneity were performed using Cochran’s Q statistic for the MR-IVW approach and Rucker’s Q statistic for the MR Egger method, both revealing significant heterogeneity (P < 0.05). The MR Egger intercept test demonstrating the absence of horizontal pleiotropy (P > 0.05). However, the MR-PRESSO test uncovered the presence of horizontal pleiotropy (P < 0.05). This was further corroborated by the identification of a significant outlier (rs115521560) and three potential outliers (rs10911902, rs3087243, rs9943599) as determined by the MR-PRESSO test. Evaluation of the MR-RAPS indicated adherence to a normal distribution (P > 0.05) (**Supplementary Table 10**). In light of the identified horizontal pleiotropy, a subsequent iteration of the MR analysis was undertaken subsequent to the exclusion of the prominent outlier. The re-evaluated random-effects IVW outcomes exhibited a positive genetic causal association between RA and urticaria (P < 0.001, OR 95% CI = 1.091 [1.051-1.133]). Beyond the elementary method, the trio of supplementary methodologies similarly detected a positive genetic causal link between RA and urticaria. Furthermore, the congruence of the three validation techniques with the outcomes of the random-effects IVW approach was affirmed (**Figures 2**, **3A**).

The outcomes of the random-effects IVW analysis revealed that SLE and urticaria not exhibited discernible genetic causality (P = 0.106, OR 95% CI = 1.024 [0.995 - 1.054]). Notably, the evaluation of genetic causal attributions unveiled substantial heterogeneity (P < 0.05). The examination of horizontal pleiotropy via the MR Egger intercept test exhibited no statistically significant evidence (P > 0.05), whereas contrasting findings arose from the MR-PRESSO assessment, indicating a noteworthy presence of horizontal pleiotropy (P < 0.05). Further analysis utilizing the MR-PRESSO methodology identified three potential outliers (rs389884, rs4388254, rs6679677). Moreover, outcomes from the MR-RAPS analysis indicated compatibility with the normal distribution assumption (P > 0.05) (**Supplementary Table 10**). Given the discerned horizontal pleiotropy, a secondary round of MR analysis was undertaken following the exclusion of the aforementioned three potential outliers. Subsequent random-effects IVW analysis demonstrated that SLE and urticaria not displayed appreciable genetic causality (P = 0.327, OR 95% CI = 1.013 [0.987 - 1.039]). These findings were consistent across four supplementary methodologies and three validation approaches, collectively indicating the absence of genetic causative underpinnings between SLE and urticaria (**Figures 2**, **3B**).

**Figure 4:**
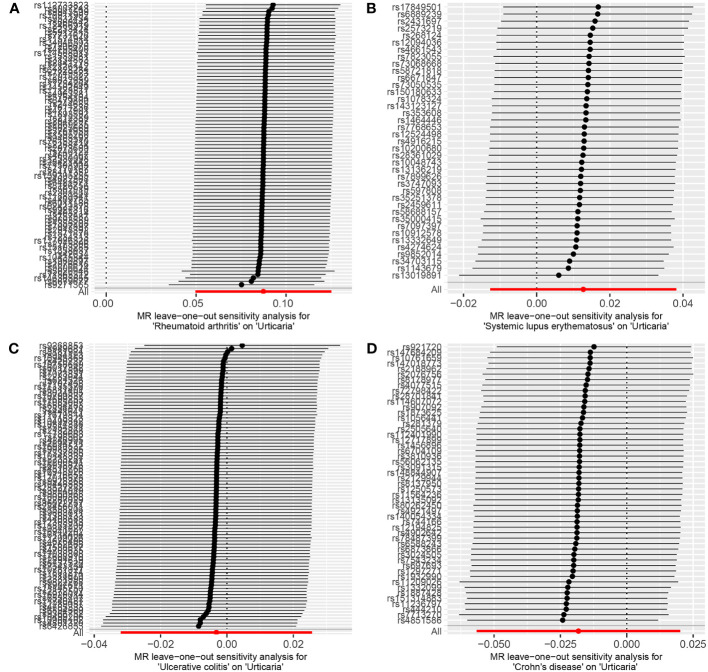
The leave-one-out analysis between exposures (RA, SLE, UC and CD) and outcome (urticaria). **(A)** RA and urticaria; **(B)** SLE and urticaria; **(C)** UC and urticaria; **(D)** CD and urticaria.

**Figure 5:**
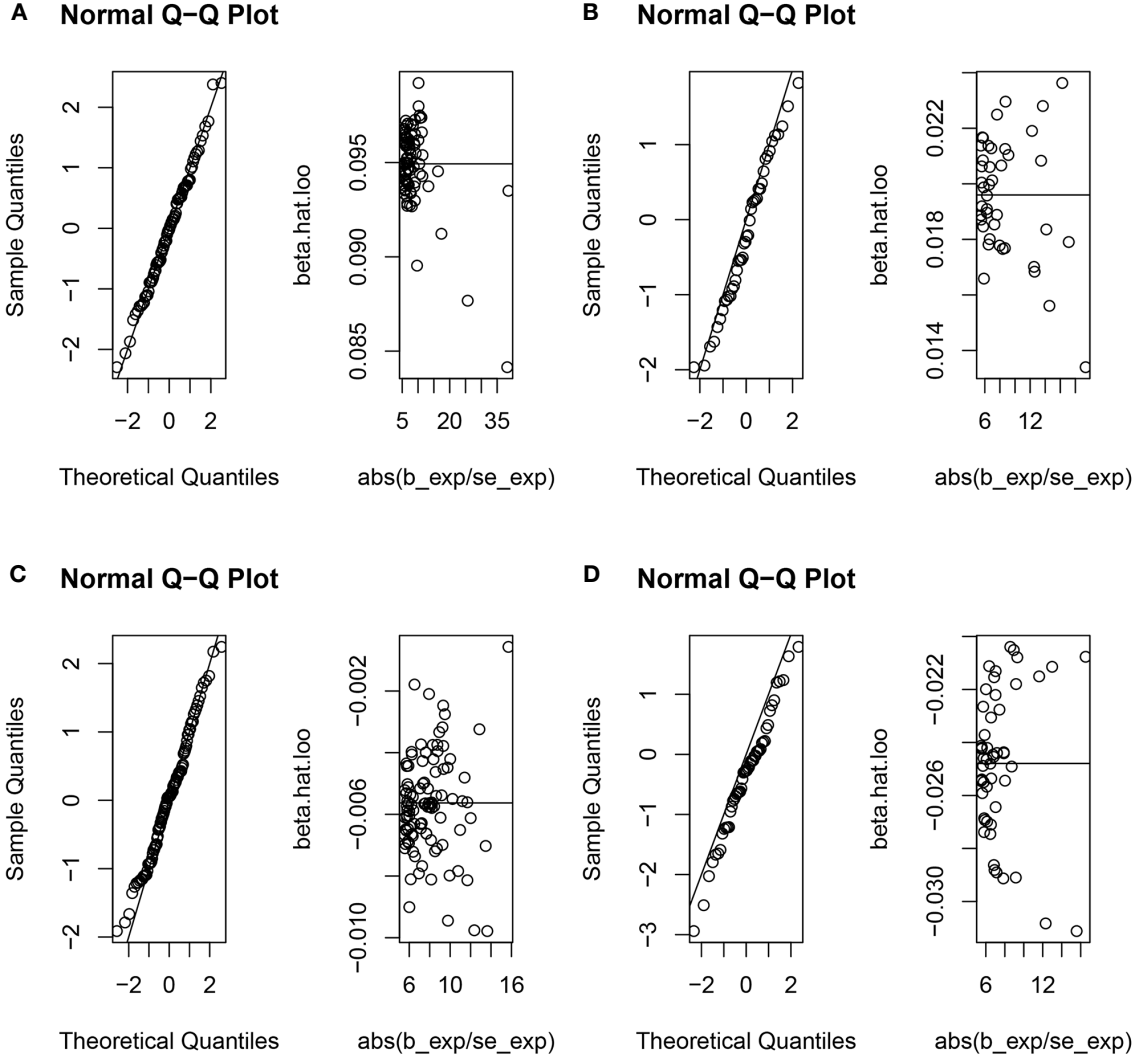
The normal distribution between exposures (RA, SLE, UC and CD) and outcome (urticaria). **(A)** RA and urticaria; **(B)** SLE and urticaria; **(C)** UC and urticaria; **(D)** CD and urticaria.

The random-effects IVW analysis revealed that there exists no significant genetic causal relationship between UC (P = 0.829, Odds OR 95% CI = 0.997 [0.968-1.026]) and CD (P = 0.352, OR 95% CI = 0.982 [0.945-1.020]) in relation to urticaria. Four supplementary analyses, as well as three validation approaches, consistently aligned with the outcomes of the random-effects IVW analysis (**Figures 2**, **3C, D**). The evaluation of genetic causality involving RA, SLE, UC, or CD, in conjunction with urticaria, exhibited uniformity in terms of the absence of heterogeneity, horizontal pleiotropy, and outliers. However, it is noteworthy that Cochran’s Q statistic for the MR-IVW analysis indicated the presence of heterogeneity within the CD and urticaria analysis (**Table 1**). Furthermore, the genetic assessment was found not affected by a single SNP (**Figure 4**), and demonstrated adherence to a normal distribution pattern (**Figure 5**, **Table 1**).

**Table 1:** Sensitivity analysis of the MR analysis results of exposures and outcomes.

Exposure	Outcome	Heterogeneity		Pleiotropy	MR-PRESSO		MR-RAPS
		Cochran’s Q Test (IVW)	Rucker’s Q Test (MR-Egger)	Intercept Test (MR-Egger)	Outliers	Pleiotropy	Normal Distribution
		P value	P value	P value	Number	P value	P value
RA	Urticaria	0.055	0.087	0.055	0	0.060	0.968
SLE	Urticaria	0.498	0.460	0.677	0	0.460	0.604
UC	Urticaria	0.614	0.592	0.626	0	0.800	0.415
CD	Urticaria	0.046	0.054	0.647	0	0.058	0.775
Urticaria	RA	0.665	0.666	0.358	0	0.227	0.973
Urticaria	SLE	0.664	0.583	0.653	0	0.238	0.179
Urticaria	UC	0.634	0.721	0.310	0	0.602	—
Urticaria	CD	0.273	0.223	0.707	0	0.233	0.876

MR, mendelian randomization; RA, rheumatoid arthritis; SLE, systemic lupus erythematosus; UC, ulcerative colitis; CD, Crohn’s disease.

### 3.2 Genetic causality between exposure (urticaria) and outcomes (RA, SLE, UC, CD)

During the course of conducting reverse MR analysis, it became evident that the number of SNPs associated with urticaria insufficient when the statistical threshold of P < 5 x 10^-8^ was applied. To address this limitation, an adjustment to the correlation threshold was implemented, leading to the adoption of a more permissive significance criterion of P < 5 x 10^-6^. As a result of this modification, a total of 19 SNPs exhibiting robust associations with urticaria were identified. Among these, 17 SNPs were sourced from the GWAS summary data of RA. No discernible SNP associations with potential confounding variables were observed. Subsequent to the exclusion of 2 SNPs (namely rs1980496 and rs205002) which were linked to RA, along with 2 palindromic SNPs (rs10925146 and rs6787175), a set of 13 SNPs was retained for use as IVs (**Supplementary Table 6**). Additionally, a cohort of 16 SNPs was garnered from GWAS summary data for SLE, whereby no SNPs associations with confounding factors. Upon the elimination of 2 SLE-associated SNPs (rs1980496 and rs205002), along with 4 palindromic SNPs (rs10925146, rs167941, rs6787175, and rs74801096), a subset of 10 SNPs was selected as IVs (**Supplementary Table 7**). Furthermore, an assemblage of 7 SNPs was identified in the context of GWAS summary data for UC, whereby no instances of SNP associations with UC or confounding factors were detected. In a parallel vein, following the exclusion of a solitary palindromic SNP (rs10925146), a collection of 6 SNPs was endorsed as IVs (**Supplementary Table 8**). Analogously, within the purview of CD, a compendium of 17 SNPs was derived from GWAS summary data, and no indications of SNP associations with CD or confounding factors were brought to light. After excluding one palindromic SNP (rs6787175), the 16 SNPs were obtained as IVs (**Supplementary Table 9**). With the sequential progression of IVs filtering elucidated herein, the schematic representation of the entire process is visually encapsulated in **Figure 1**.

**Figure 6:**
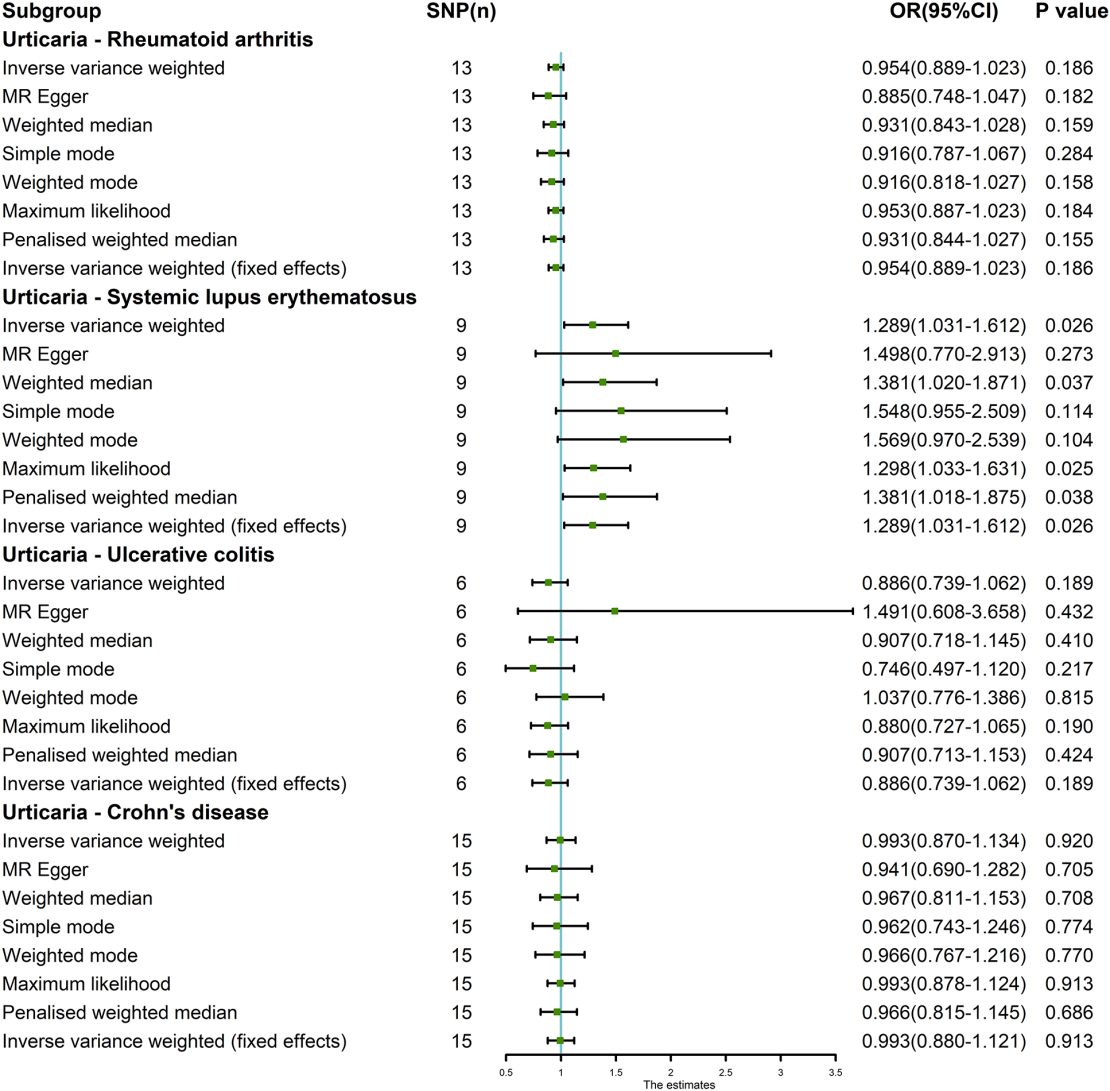
The MR analysis results of exposure (urticaria) and outcomes (RA, SLE, UC and CD). The analysis employed eight methods, namely random-effects IVW, MR Egger, weighted median, simple mode, weighted mode, maximum likelihood, penalized weighted median, and fixed-effects IVW.

**Figure 7:**
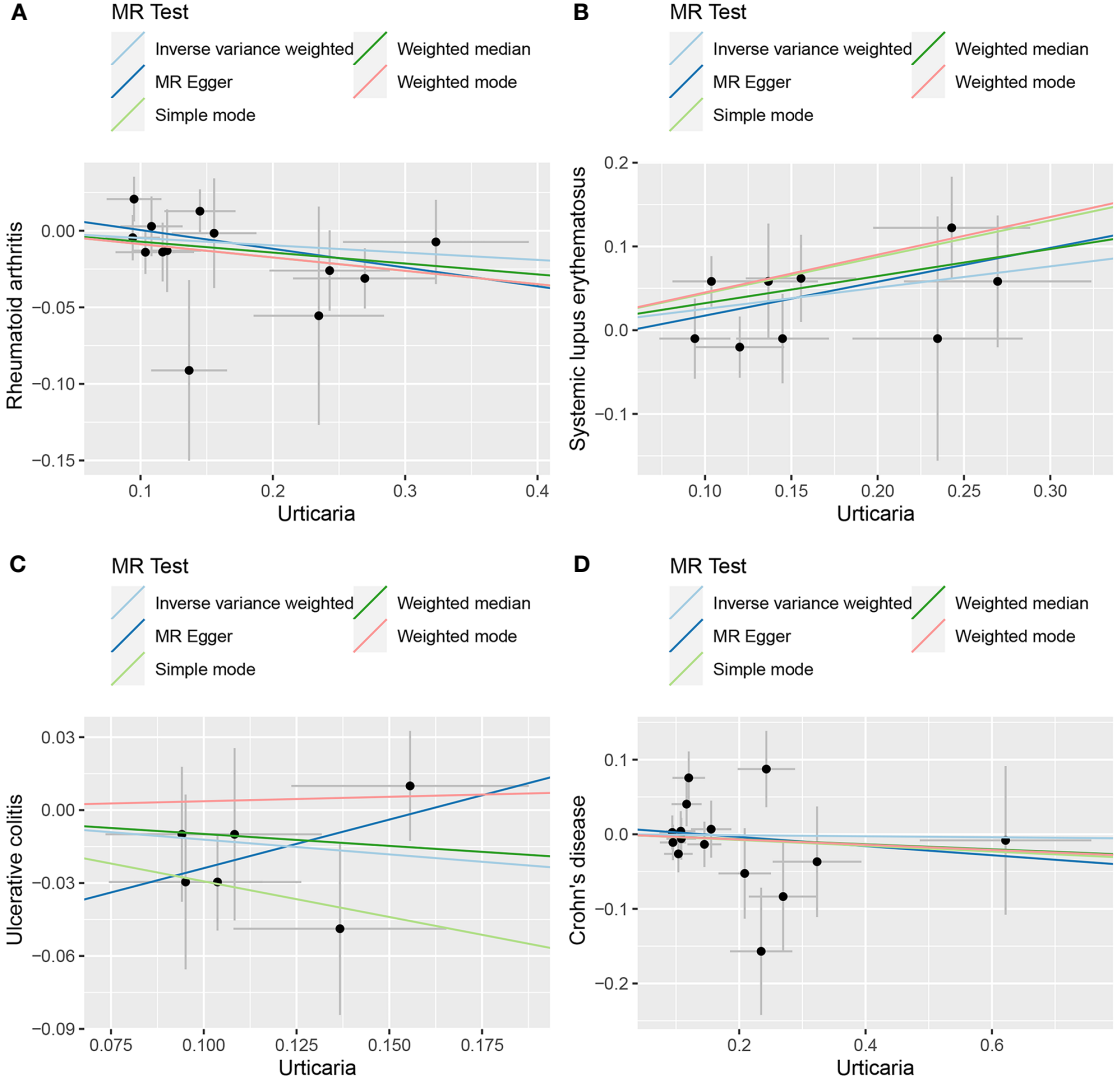
The scatter plot of MR analysis between exposure (urticaria) and outcomes (RA, SLE, UC and CD). **(A)** urticaria and RA; **(B)** urticaria and SLE; **(C)** urticaria and UC; **(D)** urticaria and CD.

The random-effects IVW approach yielded results indicating the absence of a genetically driven causal nexus between urticaria and RA (P = 0.186, OR with a 95% CI of 0.954 [0.889-1.023]). Complementary to this, four supplementary methodologies along with three validation techniques collectively corroborate this outcome (**Figures 6**, **7A**). Similarly, employing the random-effects IVW method, we ascertained that there exists no discernible genetic causal connection between urticaria and SLE (P = 0.303, OR 95% CI = 1.152 [0.880 - 1.509]). Notably, the absence of heterogeneity (P > 0.05) and the absence of horizontal pleiotropy, as determined through the intercept test of MR Egger (P > 0.05). However, it is noteworthy that the MR-PRESSO test indicated the presence of horizontal pleiotropy (P < 0.05). Furthermore, this test highlighted the presence of a significant outlier (rs4258792). Subsequently, employing the MR-RAPS analysis, we observed that the distribution of the MR analysis adheres to a normal distribution (P > 0.05) (**Supplementary Table 10**). A secondary round of MR analysis was executed after the exclusion of the aforementioned significant outlier. Intriguingly, the random-effects IVW approach revealed the presence of a positive genetic causal relationship linking urticaria with SLE (P = 0.026, OR 95% CI = 1.289 [1.031-1.612]). Among the four supplementary methodologies, the weighted median approach consistently corroborated this newfound inference. This conclusion was further supported by three additional verification methods (**Figures 6**, **7B**).

The random-effects IVW approach revealed non-significant evidence of a genetic causal association between urticaria and UC (P = 0.189, OR 95% CI = 0.886 [0.739-1.062]). This observation was corroborated by four supplementary methodologies and three validation techniques (**Figures 6**, **7C**). Similarly, employing the random-effects IVW technique, our analysis indicated the absence of a genetic causal link between urticaria and CD (P = 0.615, OR 95% CI = 0.960 [0.820 - 1.125]). Our MR investigation demonstrated heterogeneity within the data (P < 0.05). Furthermore, no significant horizontal pleiotropy was detected through the MR Egger intercept test (P > 0.05), although the MR-PRESSO test detected indications of horizontal pleiotropy (P < 0.05). This assessment also identified a significant outlier (rs9982936) and two potential outliers (rs56043070, rs652197). Additionally, MR-RAPS analysis verified the normal distribution adherence of our MR findings (P > 0.05) (**Supplementary Table 10**). A secondary round of MR analysis was conducted subsequent to the removal of a significant outlier. The random-effects IVW method yielded non-significant outcomes in terms of a genetic causal association between urticaria and CD (P = 0.920, OR 95% CI = 0.993 [0.870-1.134]). Consistent results were obtained through four supplementary approaches and three validation procedures, further supporting the absence of genetic causality between urticaria and CD (**Figures 6**, **7D**).

**Figure 8:**
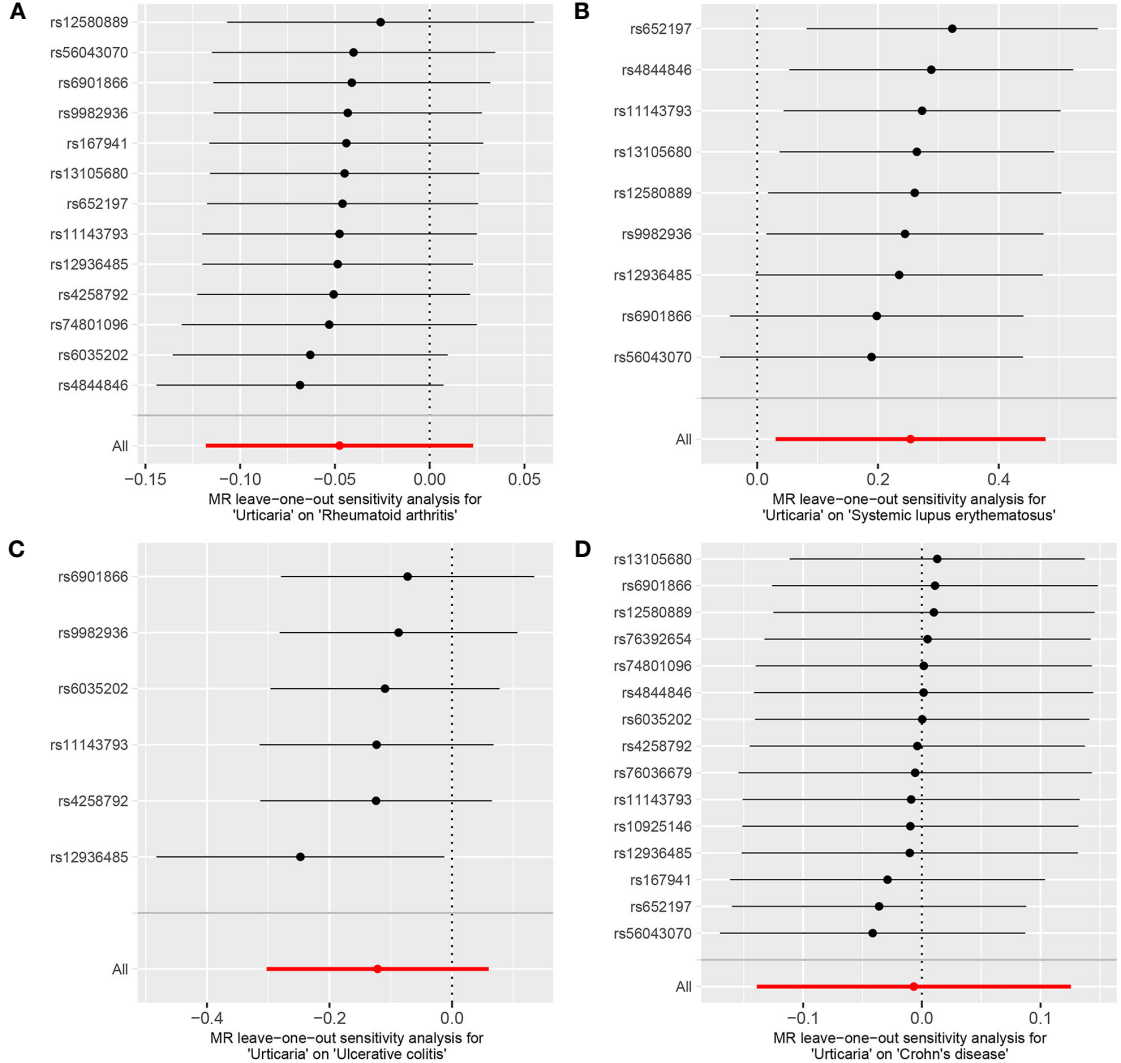
The leave-one-out analysis between exposure (urticaria) and outcomes (RA, SLE, UC and CD). **(A)** urticaria and RA; **(B)** urticaria and SLE; **(C)** urticaria and UC; **(D)** urticaria and CD.

**Figure 9:**
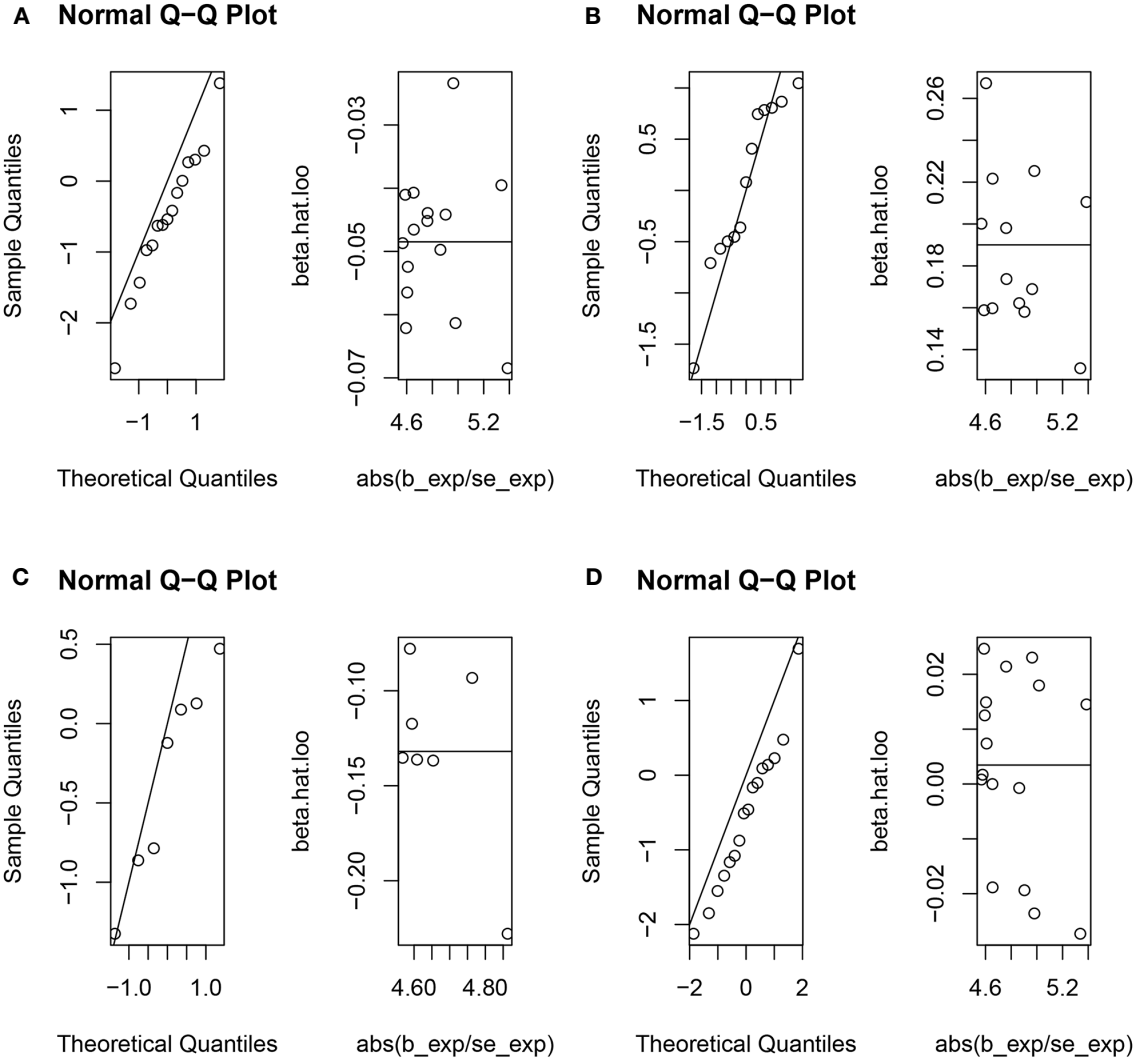
The normal distribution between exposure (urticaria) and outcomes (RA, SLE, UC and CD). **(A)** urticaria and RA; **(B)** urticaria and SLE; **(C)** urticaria and UC; **(D)** urticaria and CD.

The genetic causal evaluation pertaining to the relationship between urticaria and conditions such as RA, SLE, UC, or CD exhibits a notable absence of heterogeneity, horizontal pleiotropy, and outliers (**Table 1**). The MR analysis is not affected by a single SNP (**Figure 8**) and conforms to a normal distribution (**Figure 9**, **Table 1**). However, it should be noted that no P-value was given for the MR-RAPS analysis of urticaria and UC as the number of IVs was only six, and MR-RAPS can only give a P-value of seven or more IVs.

## 4 Discussion

This research endeavor embarked upon an examination of the inherent causal interplay existing at the genetic stratum between prevalent autoimmune disorders, namely RA, SLE, UC, CD, and urticaria. Employing a bidirectional two-sample MR analysis, compelling evidence emerged to support a positive genetic causal nexus between RA and urticaria, thereby implying the potential of RA as a predisposing factor for urticaria. Moreover, the employment of reverse MR analysis discerned a substantiated positive genetic causal relationship linking urticaria with SLE, thus implicating urticaria as a plausible risk factor for SLE. Nevertheless, no discernible positive or negative genetic causal linkage surfaced in relation to urticaria and the remaining autoimmune disorders under scrutiny.

The relationship between RA and urticaria have received limited attention within the realm of research. An isolated case report has documented the emergence of urticaria subsequent to tacrolimus administration for the treatment of RA (30). Study has exhibited a moderate level of efficacy in managing patients affected by chronic spontaneous urticaria and RA through the combined utilization of omalizumab and etanercept (31). It is notable that RA can precipitate adrenergic urticaria by the mere introduction of supplementary pressure, while it can also incite urticaria by engaging in a systematic activation of mast cells (32). Mast cells are integral to the etiology of chronic inflammatory maladies. Empirical evidence has affirmed the participation of mast cell-mediated inflammation and antibodies in the genesis of RA (33). Elevation in the mast cell density within the synovial tissue of RA-afflicted individuals has been observed in contrast to those with normal joints and osteoarthritis joints (34). Mast cells, commonly recognized in allergic pathologies, are also prevalent within RA synovial tissue and synovial fluid (35). The hyperplasia of mast cells within RA synovial tissue constitutes approximately 5% of the total synovial cell population (35). Urticaria is mainly caused by activation and degranulation of mast cells, and type II hypersensitivity promoted by IgG autoantibodies binding to IgE receptors on mast cells is considered to be the main immune pathway in the pathophysiology of urticaria. The findings of the present inquiry posit a positive and genetically driven causal association linking RA with urticaria, thereby underscoring a heightened vulnerability to urticarial manifestations among RA patients. This outcome aligns with the determinations of antecedent investigations. Our theory that the augmented proliferation of mast cells within the RA synovium engenders an escalated susceptibility to urticaria.

Urticaria and SLE exhibit a close interrelationship, with evident parallels in terms of autoimmune etiology and cutaneous presentations. Within the pathogenesis of both conditions, intricate connections exist among processes of inflammation, autoimmunity, complement activation, and coagulation, which reciprocally potentiate disease progression. Central to the initial stages of the pathogenic cascade in both urticaria and SLE are the presence of IgG and IgE autoantibodies (14). Urticaria may manifest as an antecedent symptom in SLE patients prior to the conventional clinical indicators of SLE becoming apparent (14). This association extends to cases of juvenile SLE, where chronic autoimmune urticaria has been identified as the inaugural presentation (36). Supported by a comprehensive national investigation in Taiwan, a notable link between urticaria and augmented SLE susceptibility has been established, grounded in shared mechanisms of inflammation and immune dysregulation underpinning the two maladies (37). A parallel exploration encompassing a national population-based case-control study elucidates an elevated risk of SLE in pediatric patients afflicted with urticaria, with a further escalation in risk proportionate to the frequency of urticarial episodes or the persistence of chronic urticaria (38). Within the realm of childhood-onset systemic lupus erythematosus, an extensive survey embracing 852 cases underscores a linkage between urticaria and early-onset SLE, while concurrently highlighting the correlation between urticaria and moderate to high SLE disease activity in the absence of major organ involvement (39). Urticarial vasculitis (UV) represents a rare form of small-vessel vasculitis characterized by recurrent and persistent wheal-like cutaneous lesions, which bear resemblance to those encountered in CSU. However, these UV lesions typically endure for longer than 24 hours and manifest histopathological attributes indicative of leukocytoclastic vasculitis. Serum complement levels serve as a basis for classifying UV into two principal subtypes: normocomplementemic UV (NUV) and hypocomplementemic UV (HUV). The etiology of renal involvement in UV, SLE, and proliferative glomerulonephritis is rooted in the deposition and subsequent accumulation of anti-C1q autoantibodies and immune complexes within the glomeruli of affected individuals. The alignment of neutrophils along the dermal-epidermal junction and the presence of dermal granular C4d deposition are robust indicators of HUV and its underlying association with SLE (40). Neutrophilic urticarial dermatosis (NUD) is a distinct cutaneous reaction pattern noted in autoinflammatory syndromes and SLE. NUD may manifest as a concurrent cutaneous manifestation in autoinflammatory conditions like Schnitzler syndrome, a rare disorder characterized by generalized exanthema and IgM monoclonal gammopathy, adult-onset Still disease, a rare systemic autoinflammatory disorder of undetermined origin, presenting a triad of persistent fever, polyarthritis, and rash, cryopyrin-associated periodic syndrome (CAPS), and SLE (40). Collectively, these lines of evidence collectively underscore the premise that urticaria functions as a predisposing factor for SLE. Furthermore, the current investigation extends this perspective through a genetic vantage point. Consequently, clinical should be alert that patients presenting with urticaria should be attuned to the potential for subsequent development of SLE.

A case report outlines the manifestation of autologous serum skin testing (ASST)-positive urticaria within a patient diagnosed with UC (41). ASST serves as a diagnostic measure for identifying histamine-releasing autoantibodies, with a positive result indicative of the presence of autoreactive urticaria. The manifestation of ASST-positive urticaria in a UC patient implies the potential existence of a correlation between the two diseases. Both ailments may be underpinned by immune aberrations, encompassing the hyperactivation of T cells and the emergence of autoreactive B cells that generate autoantibodies (41). An additional case report delineates the emergence of delayed pressure urticaria in a patient afflicted by UC (42). Furthermore, a documented case report expounds upon the concurrent presentation of giant urticaria and UC (43). The therapeutic intervention for UC not only mitigates colitis-related symptoms but also precipitates a swift resolution of cutaneous manifestations. This underscores the notion that urticaria could potentially serve as an incidental symptom of UC (43). Instances of CD coexisting with urticaria are similarly infrequent. A case report elucidates the co-occurrence of CD and chronic idiopathic urticaria (44). A recent publication chronicles a pediatric case involving refractory chronic spontaneous urticaria alongside CD (45). Even in patients subjected to immunosuppressive therapy, a nexus between chronic spontaneous urticaria and CD may persist, which can be effectively managed through the administration of monoclonal anti-IgE antibodies (45). While the present study did not yield conclusive evidence establishing a causal connection between UC, CD and urticaria at the genetic level, the possibility of an association at other levels beyond genetics remains open.

The present study, despite its valuable insights, is not without its inherent limitations. Primarily, it is important to acknowledge that the genomic data utilized for this investigation emanate exclusively from European cohorts. Consequently, the generalizability of the derived conclusions to cohorts of greater ethnical and genetic diversity is inherently constrained. Additionally, the scope of this study was confined to probing exclusively into the causal interplay existing between common autoimmune disorders and urticaria. Regrettably, the comprehensive spectrum of autoimmune afflictions could not be encompassed within the confines of this research endeavor. Thirdly, it is worth noting that urticaria comprises numerous distinct categories; however, there is a paucity of GWAS data available for each specific subtype within the urticaria spectrum. In light of this limitation, the present investigation undertook an analysis of GWAS data encompassing the entirety of the urticaria condition. As the field of urticaria GWAS research continues to evolve, it becomes increasingly evident that a promising avenue for future exploration lies in investigating the potential correlations between the various subtypes of urticaria and autoimmune diseases.

## 5 Conclusion

This investigation undertook an analysis of the inherent causal interconnections existing at the genetic stratum between four prevalent autoimmune disorders and urticaria. The results indicate an positive genetic causative nexus between RA and urticaria, with indications that RA could potentially serve as a predisposing factor for urticaria. Furthermore, a positive genetic causal rapport between urticaria and SLE was also established, suggesting that urticaria might exert influence as a contributing element to the susceptibility to SLE. The implications of these research findings introduce a novel vantage point pertaining to the intricate interplay between urticaria and autoimmune pathologies. It is recommended that individuals diagnosed with RA exercise heightened vigilance with regard to the manifestation of urticaria within the clinical context. Analogously, for patients grappling with urticaria, a cognizant awareness of the potential risk for SLE is advised.

## Data availability statement

The datasets presented in this study can be found in online repositories. The names of the repository/repositories and accession number(s) can be found in the article/**Supplementary Material**.

## Author contributions

MY: Data curation, Formal Analysis, Methodology, Writing – original draft, Writing – review & editing. YS: Data curation, Formal Analysis, Methodology, Writing – original draft. KX: Data curation, Formal Analysis, Methodology, Writing – original draft. PW: Data curation, Methodology, Writing – original draft. BZ: Data curation, Methodology, Writing – original draft. JG: Data curation, Methodology, Writing – original draft. KN: Software, Visualization, Writing – original draft. PY: Software, Visualization, Writing – original draft. XS: Software, Visualization, Writing – original draft. LL: Software, Visualization, Writing – original draft. ZY: Software, Visualization, Writing – original draft. PX: Conceptualization, Investigation, Project administration, Supervision, Writing – review & editing.

## References

[1] Poonawalla T Kelly B. Urticaria : a review. Am J Clin Dermatol (2009) 10(1):9–21. doi: 10.2165/0128071-200910010-00002 19170406

[2] Kolkhir P Giménez-Arnau AM Kulthanan K Peter J Metz M Maurer M. Urticaria. Nat Rev Dis Primers. (2022) 8(1):61. doi: 10.1038/s41572-022-00389-z 36109590

[3] Wedi B Wieczorek D Raap U Kapp A. Urticaria. JDDG: J der Deutschen Dermatologischen Gesellschaft. (2014) 12(11):997–1009. doi: 10.1111/ddg.12441 25273223

[4] Greaves M. Autoimmune urticaria. Clin Rev Allergy Immunol (2002) 23(2):171–83. doi: 10.1385/CRIAI:23:2:171 12221862

[5] Wedi B. Urticaria. Jddg (2008) 6(4):306–17. doi: 10.1111/j.1610-0387.2008.06661.x 18377563

[6] Gonçalo M Gimenéz-Arnau A Al-Ahmad M Ben-Shoshan M Bernstein JA Ensina LF. The global burden of chronic urticaria for the patient and society*. Br J Dermatol (2020) 184(2):226–36. doi: 10.1111/bjd.19561 32956489

[7] Fricke J Ávila G Keller T Weller K Lau S Maurer M. Prevalence of chronic urticaria in children and adults across the globe: Systematic review with meta-analysis. Allergy (2019) 75(2):423–32. doi: 10.1111/all.14037 31494963

[8] Choi G-S Nam Y-H Park C-S Kim M-Y Jo E-J Park H-K. Anxiety, depression, and stress in Korean patients with chronic urticaria. Korean J Internal Med (2020) 35(6):1507–16. doi: 10.3904/kjim.2019.320 PMC7652653 32450676

[9] Sánchez-Díaz M Salazar-Nievas M-C Molina-Leyva A Arias-Santiago S. Type D personality is associated with poorer quality of life in patients with chronic spontaneous urticaria: A cross-sectional study. Acta Dermato-Venereologica. (2022) 102:adv00734. doi: 10.2340/actadv.v102.676 35470405 PMC9631268

[10] Hennino A Bérard F Guillot I Saad N Rozières A Nicolas J-F. Pathophysiology of urticaria. Clin Rev Allergy Immunol (2006) 30(1):3–11. doi: 10.1385/CRIAI:30:1:003 16461989

[11] Maronese CA Ferrucci SM Moltrasio C Lorini M Carbonelli V Asero R. IgG and igE autoantibodies to IgE receptors in chronic spontaneous urticaria and their role in the response to omalizumab. J Clin Med (2023) 12(1):378. doi: 10.3390/jcm12010378 36615181 PMC9821397

[12] Kolkhir P Muñoz M Asero R Ferrer M Kocatürk E Metz M. Autoimmune chronic spontaneous urticaria. J Allergy Clin Immunol (2022) 149(6):1819–31. doi: 10.1016/j.jaci.2022.04.010 35667749

[13] Stitt JM Dreskin SC. Urticaria and autoimmunity: where are we now? Curr Allergy Asthma Rep (2013) 13(5):555–62. doi: 10.1007/s11882-013-0366-8 23821106

[14] Kolkhir P Pogorelov D Olisova O Maurer M. Comorbidity and pathogenic links of chronic spontaneous urticaria and systemic lupus erythematosus - a systematic review. Clin Exp Allergy (2016) 46(2):275–87. doi: 10.1111/cea.12673 26545308

[15] Kolkhir P Borzova E Grattan C Asero R Pogorelov D Maurer M. Autoimmune comorbidity in chronic spontaneous urticaria: A systematic review. Autoimmun Rev (2017) 16(12):1196–208. doi: 10.1016/j.autrev.2017.10.003 29037900

[16] Darlenski R Kazandjieva J Zuberbier T Tsankov N. Chronic urticaria as a systemic disease. Clinics Dermatol (2014) 32(3):420–3. doi: 10.1016/j.clindermatol.2013.11.009 24767190

[17] Chiu H-Y Muo C-H Sung F-C. Associations of chronic urticaria with atopic and autoimmune comorbidities: a nationwide population-based study. Int J Dermatol (2018) 57(7):822–9. doi: 10.1111/ijd.14000 29663342

[18] Hu S Xing H Wang X Zhang N Xu Q. Causal relationships between total physical activity and ankylosing spondylitis: A mendelian randomization study. Front Immunol (2022) 13:887326. doi: 10.3389/fimmu.2022.887326 35865535 PMC9294357

[19] Shi YZ Tao QF Qin HY Li Y Zheng H. Causal relationship between gut microbiota and urticaria: a bidirectional two-sample mendelian randomization study. Front Microbiol (2023) 14:1189484. doi: 10.3389/fmicb.2023.1189484 37426010 PMC10324650

[20] Sabroe RA. Acute urticaria. Immunol Allergy Clin North Am (2014) 34(1):11–21. doi: 10.1016/j.iac.2013.07.010 24262686

[21] Kim YH Do Han K Bang CH Lee JH Lee JY Park YG. High waist circumference rather than high body mass index may be a predictive risk factor for the longer disease duration of chronic spontaneous urticaria. Sci Rep (2021) 11(1):1875. doi: 10.1038/s41598-021-81484-1 33479357 PMC7820591

[22] Petrovska N Prajzlerova K Vencovsky J Senolt L Filkova M. The pre-clinical phase of rheumatoid arthritis: From risk factors to prevention of arthritis. Autoimmun Rev (2021) 20(5):102797. doi: 10.1016/j.autrev.2021.102797 33746022

[23] Qin Q Zhao L Ren A Li W Ma R Peng Q. Systemic lupus erythematosus is causally associated with hypothyroidism, but not hyperthyroidism: A Mendelian randomization study. Front Immunol (2023) 14:1125415. doi: 10.3389/fimmu.2023.1125415 36860870 PMC9968792

[24] Ordas I Eckmann L Talamini M Baumgart DC Sandborn WJ. Ulcerative colitis. Lancet (London England) (2012) 380(9853):1606–19. doi: 10.1016/S0140-6736(12)60150-0 22914296

[25] Torres J Mehandru S Colombel JF Peyrin-Biroulet L. Crohn's disease. Lancet (London England). (2017) 389(10080):1741–55. doi: 10.1016/S0140-6736(16)31711-1 27914655

[26] Cai Y Zhang G Liang J Jing Z Zhang R Lv L. Causal relationships between osteoarthritis and senile central nerve system dysfunction: A bidirectional two-sample mendelian randomization study. Front Aging Neurosci (2021) 13:793023. doi: 10.3389/fnagi.2021.793023 35317304 PMC8934417

[27] Lee YH. Causal association between smoking behavior and the decreased risk of osteoarthritis: a Mendelian randomization. Z Rheumatol (2019) 78(5):461–6. doi: 10.1007/s00393-018-0505-7 29974223

[28] Cao Z Wu Y Li Q Li Y Wu J. A causal relationship between childhood obesity and risk of osteoarthritis: results from a two-sample Mendelian randomization analysis. Ann Med (2022) 54(1):1636–45. doi: 10.1080/07853890.2022.2085883 PMC9225762 35703935

[29] Yang M Xu J Zhang F Luo P Xu K Feng R. Large-scale genetic correlation analysis between spondyloarthritis and human blood metabolites. J Clin Med (2023) 12(3):1201. doi: 10.3390/jcm12031201 36769847 PMC9917834

[30] Kamata Y Iwamoto M Minota S. Treatment of rheumatoid arthritis with tacrolimus: tacrolimus-induced urticaria. J Clin Rheumatol (2009) 15(4):213. doi: 10.1097/RHU.0b013e3181a7b0ec 19502910

[31] Ghazanfar MN Thomsen SF. Combined treatment with omalizumab and etanercept in a patient with chronic spontaneous urticaria and rheumatoid arthritis. J Dermatol Treat (2018) 30(4):387–8. doi: 10.1080/09546634.2018.1515465 30132352

[32] Capella GL. Adrenergic urticaria and rheumatoid arthritis in a patient with melanoma: an intricate medical management. J Drugs Dermatol (2012) 11(3):409–12. 22395595

[33] Kritas SK Saggini A Varvara G Murmura G Caraffa A Antinolfi P. Mast cell involvement in rheumatoid arthritis. J Biol Regul Homeost Agents. (2013) 27(3):655–60. 24152834

[34] Gotis-Graham I Smith MD Parker A McNeil HP. Synovial mast cell responses during clinical improvement in early rheumatoid arthritis. Ann rheumatic diseases. (1998) 57(11):664–71. doi: 10.1136/ard.57.11.664 PMC1752502 9924208

[35] Kim K-W Kim B-M Won J-Y Min H-K Lee K-A Lee S-H. Regulation of osteoclastogenesis by mast cell in rheumatoid arthritis. Arthritis Res Ther (2021) 23(1):124. doi: 10.1186/s13075-021-02491-1 33882986 PMC8059019

[36] Spadoni M Jacob C Aikawa N Jesus A Fomin A Silva C. Chronic autoimmune urticaria as the first manifestation of juvenile systemic lupus erythematosus. Lupus (2011) 20(7):763–6. doi: 10.1177/0961203310392428 21183563

[37] Yong S-B Su K-W Chen H-H Huang J-Y Wu H-J Wei JC-C. Impact of chronic urticaria on systemic lupus erythematosus: A nationwide population-based study in Taiwan. J Dermatol (2019) 46(1):26–32. doi: 10.1111/1346-8138.14683 30368876

[38] Lin C-H Hung P-H Hu H-Y Chung C-J Chen T-H Hung K-Y. Clinically diagnosed urticaria and risk of systemic lupus erythematosus in children: A nationwide population-based case-control study. Pediatr Allergy Immunol (2018) 29(7):732–9. doi: 10.1111/pai.12962 30054929

[39] Ferriani MPL Silva MFC Pereira RMR Terreri MT Saad Magalhães C Bonfá E. Chronic spontaneous urticaria: A survey of 852 cases of childhood-onset systemic lupus erythematosus. Int Arch Allergy Immunol (2015) 167(3):186–92. doi: 10.1159/000438723 26329010

[40] Marzano AV Maronese CA Genovese G Ferrucci S Moltrasio C Asero R. Urticarial vasculitis: Clinical and laboratory findings with a particular emphasis on differential diagnosis. J Allergy Clin Immunol (2022) 149(4):1137–49. doi: 10.1016/j.jaci.2022.02.007 35396080

[41] Tedeschi A Lorini M Airaghi L. Chronic autoreactive urticaria in a patient with ulcerative colitis. J Clin Gastroenterol (2003) 36(5):454–5. doi: 10.1097/00004836-200305000-00023 12702996

[42] Hassiko H Guilchard FL Nembo J Lespessailles E Martin L Benhamou CL. Delayed pressure urticaria in a patient with ulcerative colitis. Joint Bone Spine. (2002) 69(5):519–20. doi: 10.1016/S1297-319X(02)00442-6 12477241

[43] Caroselli C Plocco M Pratticò F Bruno C Antonaglia C Rota F. Ulcerative colitis masked by giant urticaria. Int J Immunopathol Pharmacol (2007) 20(1):181–4. doi: 10.1177/039463200702000121 17346442

[44] Witten J Siles R Shen B Yao Q. Triple disease combination. Inflammatory Bowel Diseases. (2016) 22(3):E12–E3. doi: 10.1097/MIB.0000000000000702 26829407

[45] Barni S Giovannini M Liccioli G Sarti L Gissi A Lionetti P. Case report: refractory chronic spontaneous urticaria treated with omalizumab in an adolescent with Crohn’s disease. Front Immunol (2021) 12. doi: 10.3389/fimmu.2021.635069 PMC7962273 33737936

