# Supplementary material for: Common autoimmune diseases and urticaria: the causal relationship from a bidirectional two-sample mendelian randomization study
Source: Front Immunol. 2023 Nov 2;14:1280135. doi: 10.3389/fimmu.2023.1280135 (PMC10652397; doi:10.3389/fimmu.2023.1280135)
Supplement: Supplementary file 1 [file Table_1.doc]

**Supplementary Table 1:** The data used in this study.

| **GWAS ID** | **Trait** | **Samples** | **SNPs** | **Population** |
| --- | --- | --- | --- | --- |
| ebi-a-GCST90013534 | Rheumatoid arthritis | 58,284 | 13,108,512 | European |
| ebi-a-GCST003156 | Systemic lupus erythematosus | 14,267 | 7,071,163 | European |
| ebi-a-GCST000964 | Ulcerative colitis | 26,405 | 1,116,795 | European |
| ieu-a-30 | Crohn's disease | 20,883 | 12,276,506 | European |
| finn-b-L12_URTICARIA | Urticaria | 217,530 | 16,380,466 | European |

**Supplementary Table 2:** The instrumental variables used in MR analysis between exposure (rheumatoid arthritis) and outcome (urticaria).

|  | **SNP** | **beta.exposure** | **se.exposure** | **pval.exposure** | **pval.outcome** | **palindromic** | **R2** | **F** |
| --- | --- | --- | --- | --- | --- | --- | --- | --- |
| 1 | rs10435844 | -0.0784 | 0.0121 | 9.73E-11 | 0.05332 | FALSE | 0.00072 | 41.98039 |
| 2 | rs10911902 | -0.0847 | 0.0152 | 2.36E-08 | 0.01408 | FALSE | 0.000532 | 31.05022 |
| 3 | rs11123811 | -0.0995 | 0.0114 | 2.01E-18 | 0.8024 | FALSE | 0.001305 | 76.17659 |
| 4 | rs112733823 | 0.191 | 0.0188 | 3.82E-24 | 0.09769 | FALSE | 0.001768 | 103.2134 |
| 5 | rs114508013 | 0.488 | 0.0441 | 1.82E-28 | 0.755899 | FALSE | 0.002097 | 122.4468 |
| 6 | rs115521560 | 0.7878 | 0.0364 | 1.29E-103 | 0.094519 | FALSE | 0.007973 | 468.3972 |
| 7 | rs11574914 | 0.1153 | 0.0149 | 9.92E-15 | 0.3898 | FALSE | 0.001026 | 59.87854 |
| 8 | rs117026326 | 0.381 | 0.0424 | 2.45E-19 | 0.1983 | FALSE | 0.001383 | 80.74271 |
| 9 | rs11754264 | -0.1359 | 0.0193 | 1.88E-12 | 0.1724 | FALSE | 0.00085 | 49.58033 |
| 10 | rs11889341 | 0.1466 | 0.0129 | 4.32E-30 | 0.3852 | FALSE | 0.002211 | 129.1438 |
| 11 | rs12126142 | -0.0751 | 0.0116 | 1.01E-10 | 0.3179 | FALSE | 0.000719 | 41.91302 |
| 12 | rs1234313 | 0.0797 | 0.0133 | 1.90E-09 | 0.1276 | FALSE | 0.000616 | 35.9086 |
| 13 | rs12466919 | 0.1025 | 0.0152 | 1.59E-11 | 0.2761 | FALSE | 0.00078 | 45.47217 |
| 14 | rs12530098 | 0.1382 | 0.0204 | 1.35E-11 | 0.7527 | FALSE | 0.000787 | 45.89241 |
| 15 | rs12918327 | 0.0867 | 0.0157 | 3.04E-08 | 0.4824 | FALSE | 0.000523 | 30.49467 |
| 16 | rs13103285 | 0.0989 | 0.0131 | 4.29E-14 | 0.1506 | FALSE | 0.000977 | 56.99478 |
| 17 | rs1355208 | 0.0818 | 0.0119 | 6.77E-12 | 0.2202 | FALSE | 0.00081 | 47.24956 |
| 18 | rs139395255 | 0.3833 | 0.0235 | 8.68E-60 | 0.5629 | FALSE | 0.004544 | 266.0278 |
| 19 | rs146305655 | -0.4379 | 0.0452 | 3.29E-22 | 0.004764 | FALSE | 0.001608 | 93.85515 |
| 20 | rs1538981 | 0.0671 | 0.0114 | 4.42E-09 | 0.2236 | FALSE | 0.000594 | 34.64339 |
| 21 | rs1571878 | -0.1539 | 0.0116 | 4.13E-40 | 0.2344 | FALSE | 0.003011 | 176.0137 |
| 22 | rs1595260 | 0.0845 | 0.0126 | 2.29E-11 | 0.9021 | FALSE | 0.000771 | 44.97358 |
| 23 | rs1611236 | -0.1165 | 0.0131 | 4.54E-19 | 0.8722 | FALSE | 0.001355 | 79.08504 |
| 24 | rs1858037 | -0.1012 | 0.0131 | 1.14E-14 | 0.1384 | FALSE | 0.001023 | 59.67653 |
| 25 | rs1883832 | 0.1052 | 0.0127 | 1.13E-16 | 0.2512 | FALSE | 0.001176 | 68.61343 |
| 26 | rs1893592 | -0.0976 | 0.0132 | 1.48E-13 | 0.8849 | FALSE | 0.000937 | 54.66846 |
| 27 | rs1950897 | 0.1069 | 0.0144 | 1.02E-13 | 0.04909 | FALSE | 0.000945 | 55.10811 |
| 28 | rs2069235 | 0.1296 | 0.014 | 1.69E-20 | 0.776599 | FALSE | 0.001468 | 85.69175 |
| 29 | rs2073609 | 0.1029 | 0.0182 | 1.47E-08 | 0.8748 | FALSE | 0.000548 | 31.96488 |
| 30 | rs2076616 | -0.0885 | 0.0135 | 6.20E-11 | 0.02568 | FALSE | 0.000737 | 42.97383 |
| 31 | rs212389 | 0.1058 | 0.0147 | 6.66E-13 | 0.9703 | FALSE | 0.000888 | 51.79905 |
| 32 | rs2233424 | 0.1964 | 0.0187 | 6.49E-26 | 0.293 | FALSE | 0.001889 | 110.3024 |
| 33 | rs2258734 | -0.0921 | 0.0123 | 6.04E-14 | 0.784899 | FALSE | 0.000961 | 56.0653 |
| 34 | rs2275806 | -0.0725 | 0.0122 | 2.51E-09 | 0.09238 | FALSE | 0.000606 | 35.31356 |
| 35 | rs2301888 | -0.1282 | 0.0121 | 3.75E-26 | 0.4115 | FALSE | 0.001922 | 112.251 |
| 36 | rs244685 | -0.089 | 0.0144 | 6.04E-10 | 0.7067 | FALSE | 0.000655 | 38.19796 |
| 37 | rs28411352 | 0.0914 | 0.0136 | 1.66E-11 | 0.574 | FALSE | 0.000774 | 45.16476 |
| 38 | rs2841275 | 0.1617 | 0.0179 | 1.71E-19 | 0.9151 | FALSE | 0.001398 | 81.60168 |
| 39 | rs28421442 | -0.1234 | 0.0214 | 7.86E-09 | 0.8278 | FALSE | 0.00057 | 33.24971 |
| 40 | rs2847297 | 0.0903 | 0.0119 | 2.65E-14 | 0.9849 | FALSE | 0.000987 | 57.57934 |
| 41 | rs2918392 | 0.0668 | 0.0122 | 4.62E-08 | 0.1378 | FALSE | 0.000514 | 29.97908 |
| 42 | rs3025669 | -0.2534 | 0.0222 | 2.91E-30 | 0.7694 | FALSE | 0.00223 | 130.2844 |
| 43 | rs3087243 | -0.1261 | 0.0124 | 3.32E-24 | 0.0218 | FALSE | 0.001771 | 103.4122 |
| 44 | rs3134883 | 0.0991 | 0.0125 | 1.98E-15 | 0.5559 | FALSE | 0.001077 | 62.85103 |
| 45 | rs34046593 | 0.1422 | 0.017 | 7.17E-17 | 0.7151 | FALSE | 0.001199 | 69.9659 |
| 46 | rs34502849 | -0.0851 | 0.014 | 1.07E-09 | 0.5627 | FALSE | 0.000634 | 36.94776 |
| 47 | rs34536443 | -0.3801 | 0.0474 | 1.08E-15 | 0.1663 | TRUE | 0.001102 | 64.30195 |
| 48 | rs3757387 | 0.1236 | 0.0137 | 1.87E-19 | 0.286 | FALSE | 0.001395 | 81.39185 |
| 49 | rs3761959 | 0.0744 | 0.0115 | 9.64E-11 | 0.9348 | FALSE | 0.000718 | 41.85384 |
| 50 | rs3806624 | 0.0863 | 0.0131 | 3.94E-11 | 0.0105 | FALSE | 0.000744 | 43.39744 |
| 51 | rs403214 | -0.0914 | 0.0146 | 3.96E-10 | 0.2875 | FALSE | 0.000672 | 39.18969 |
| 52 | rs42034 | 0.0871 | 0.0153 | 1.28E-08 | 0.08741 | FALSE | 0.000556 | 32.40698 |
| 53 | rs4409785 | 0.0982 | 0.017 | 7.85E-09 | 0.3556 | FALSE | 0.000572 | 33.36647 |
| 54 | rs4602367 | 0.075 | 0.0117 | 1.76E-10 | 0.7621 | FALSE | 0.000705 | 41.08998 |
| 55 | rs4622308 | 0.0878 | 0.0125 | 2.21E-12 | 0.5521 | FALSE | 0.000846 | 49.33488 |
| 56 | rs4717901 | 0.249 | 0.0349 | 9.52E-13 | 0.8741 | FALSE | 0.000873 | 50.90178 |
| 57 | rs4795400 | 0.0743 | 0.012 | 5.86E-10 | 0.3113 | FALSE | 0.000657 | 38.33542 |
| 58 | rs4963581 | 0.0856 | 0.0156 | 3.75E-08 | 0.4784 | FALSE | 0.000516 | 30.10811 |
| 59 | rs5020946 | 0.6519 | 0.0169 | 1.00E-200 | 0.01019 | FALSE | 0.024894 | 1487.9 |
| 60 | rs502919 | 0.0829 | 0.0134 | 6.17E-10 | 0.4545 | FALSE | 0.000656 | 38.2723 |
| 61 | rs5754104 | 0.0891 | 0.0139 | 1.36E-10 | 0.7347 | FALSE | 0.000704 | 41.08761 |
| 62 | rs5912815 | -0.0787 | 0.0134 | 4.79E-09 | 0.2196 | FALSE | 0.000591 | 34.49252 |
| 63 | rs6011186 | -0.1074 | 0.0171 | 3.19E-10 | 0.3005 | FALSE | 0.000676 | 39.44586 |
| 64 | rs61828284 | -0.2018 | 0.0348 | 6.33E-09 | 0.719999 | FALSE | 0.000577 | 33.62551 |
| 65 | rs62422878 | 0.1037 | 0.0176 | 3.57E-09 | 0.3321 | FALSE | 0.000595 | 34.71501 |
| 66 | rs6421571 | 0.134 | 0.0178 | 5.57E-14 | 0.5241 | FALSE | 0.000971 | 56.67019 |
| 67 | rs6479800 | 0.1202 | 0.0181 | 3.01E-11 | 0.8621 | FALSE | 0.000756 | 44.09983 |
| 68 | rs660442 | -0.1067 | 0.0175 | 1.11E-09 | 0.2068 | FALSE | 0.000637 | 37.17388 |
| 69 | rs6679677 | 0.591 | 0.023 | 1.41E-145 | 0.005784 | FALSE | 0.011202 | 660.2439 |
| 70 | rs7097397 | -0.0847 | 0.012 | 1.42E-12 | 0.2155 | FALSE | 0.000854 | 49.81836 |
| 71 | rs71508903 | 0.1487 | 0.0143 | 3.13E-25 | 0.5952 | FALSE | 0.001852 | 108.1272 |
| 72 | rs71565312 | 0.699 | 0.0402 | 1.11E-67 | 0.02382 | FALSE | 0.005161 | 302.3342 |
| 73 | rs7170107 | 0.1366 | 0.0158 | 6.11E-18 | 0.532 | FALSE | 0.001281 | 74.74331 |
| 74 | rs7206670 | 0.0701 | 0.0119 | 4.14E-09 | 0.1802 | FALSE | 0.000595 | 34.69982 |
| 75 | rs740122 | -0.0782 | 0.0134 | 5.37E-09 | 0.8258 | FALSE | 0.000584 | 34.05564 |
| 76 | rs76153210 | 0.1597 | 0.0205 | 6.83E-15 | 0.863 | FALSE | 0.00104 | 60.68582 |
| 77 | rs7731626 | -0.1956 | 0.0184 | 1.94E-26 | 0.9803 | FALSE | 0.001935 | 113.0023 |
| 78 | rs7749323 | 0.2835 | 0.0253 | 3.47E-29 | 0.4782 | FALSE | 0.00215 | 125.5597 |
| 79 | rs8032939 | 0.1244 | 0.0123 | 4.47E-24 | 0.8727 | FALSE | 0.001752 | 102.2859 |
| 80 | rs8126756 | -0.0823 | 0.0137 | 1.81E-09 | 0.5346 | FALSE | 0.000619 | 36.08641 |
| 81 | rs9271365 | 0.4888 | 0.0128 | 1.00E-200 | 0.000413 | FALSE | 0.02441 | 1458.235 |
| 82 | rs9405192 | -0.089 | 0.0137 | 9.26E-11 | 0.8616 | FALSE | 0.000724 | 42.20112 |
| 83 | rs9532434 | 0.114 | 0.0126 | 1.94E-19 | 0.333 | FALSE | 0.001403 | 81.8566 |
| 84 | rs9693589 | 0.1127 | 0.0128 | 1.50E-18 | 0.2409 | FALSE | 0.001328 | 77.51986 |
| 85 | rs9927316 | 0.0906 | 0.0136 | 2.30E-11 | 0.2673 | FALSE | 0.000761 | 44.37759 |
| 86 | rs9943599 | 0.083 | 0.0131 | 2.70E-10 | 0.0173 | FALSE | 0.000688 | 40.14197 |
| Confounder | |  | | | | | | |
| 1 | rs1883832 | Chronic hepatitis B infection | | | | | | |

**Supplementary Table 3:** The instrumental variables used in MR analysis between exposure (systemic lupus erythematosus) and outcome (urticaria).

|  | **SNP** | **beta.exposure** | **se.exposure** | **pval.exposure** | **pval.outcome** | **palindromic** | **R2** | **F** |
| --- | --- | --- | --- | --- | --- | --- | --- | --- |
| 1 | rs10048743 | -0.23111 | 0.041206 | 2.04E-08 | 0.6949 | FALSE | 0.0022 | 31.45369 |
| 2 | rs10200680 | -0.24846 | 0.042484 | 4.96E-09 | 0.9707 | FALSE | 0.002392 | 34.19913 |
| 3 | rs1078324 | -0.71335 | 0.078167 | 7.11E-20 | 0.8338 | FALSE | 0.005804 | 83.27279 |
| 4 | rs10912578 | -0.24686 | 0.030992 | 1.65E-15 | 0.3034 | FALSE | 0.004427 | 63.43763 |
| 5 | rs1143679 | 0.582216 | 0.039987 | 5.03E-48 | 0.1388 | FALSE | 0.014642 | 211.972 |
| 6 | rs12094036 | -0.3285 | 0.05786 | 1.37E-08 | 0.2873 | FALSE | 0.002254 | 32.2308 |
| 7 | rs12524498 | -0.67335 | 0.120793 | 2.48E-08 | 0.9604 | FALSE | 0.002173 | 31.06926 |
| 8 | rs13019891 | -0.56212 | 0.029034 | 1.65E-83 | 0.1042 | FALSE | 0.025601 | 374.7951 |
| 9 | rs13136219 | -0.17435 | 0.027787 | 3.50E-10 | 0.6469 | FALSE | 0.002752 | 39.3654 |
| 10 | rs13332649 | -0.31471 | 0.037568 | 5.43E-17 | 0.3434 | FALSE | 0.004895 | 70.16489 |
| 11 | rs143123127 | 0.470004 | 0.084034 | 2.23E-08 | 0.7212 | FALSE | 0.002188 | 31.27736 |
| 12 | rs1464446 | -0.3285 | 0.04015 | 2.79E-16 | 0.8655 | FALSE | 0.00467 | 66.93539 |
| 13 | rs150180633 | 0.928219 | 0.068957 | 2.66E-41 | 0.2725 | FALSE | 0.012541 | 181.1672 |
| 14 | rs17849501 | 0.81093 | 0.049864 | 1.81E-59 | 0.1842 | FALSE | 0.0182 | 264.4406 |
| 15 | rs2431697 | -0.22314 | 0.029296 | 2.60E-14 | 0.1068 | FALSE | 0.00405 | 58.00708 |
| 16 | rs2459611 | 0.261365 | 0.045245 | 7.62E-09 | 0.247 | FALSE | 0.002333 | 33.36513 |
| 17 | rs2573219 | 0.587787 | 0.042929 | 1.13E-42 | 0.4819 | FALSE | 0.01297 | 187.4448 |
| 18 | rs268124 | 0.18633 | 0.03237 | 8.60E-09 | 0.2488 | FALSE | 0.002317 | 33.12922 |
| 19 | rs2736332 | 0.277632 | 0.032069 | 4.83E-18 | 0.261 | TRUE | 0.005226 | 74.93703 |
| 20 | rs28361029 | -0.38566 | 0.06136 | 3.27E-10 | 0.8639 | FALSE | 0.002761 | 39.49812 |
| 21 | rs28361029 | -0.38566 | 0.06136 | 3.27E-10 | 0.138 | TRUE | 0.002761 | 39.49812 |
| 22 | rs28834423 | 0.457425 | 0.03653 | 5.65E-36 | 0.2523 | TRUE | 0.010871 | 156.7803 |
| 23 | rs34703115 | -0.61619 | 0.104778 | 4.08E-09 | 0.02944 | FALSE | 0.002418 | 34.57981 |
| 24 | rs35000415 | 0.587787 | 0.041539 | 1.86E-45 | 0.5023 | FALSE | 0.01384 | 200.2013 |
| 25 | rs35251378 | -0.23572 | 0.032427 | 3.61E-13 | 0.4793 | FALSE | 0.00369 | 52.8368 |
| 26 | rs353608 | 0.18633 | 0.02802 | 2.93E-11 | 0.722199 | FALSE | 0.00309 | 44.21551 |
| 27 | rs3747093 | 0.262364 | 0.034506 | 2.88E-14 | 0.604901 | FALSE | 0.004036 | 57.80574 |
| 28 | rs389884 | 0.928219 | 0.043232 | 2.92E-102 | 0.000863 | FALSE | 0.0313 | 460.9262 |
| 29 | rs4274624 | -0.55962 | 0.032679 | 9.73E-66 | 0.4414 | FALSE | 0.020141 | 293.2103 |
| 30 | rs4388254 | 0.378436 | 0.060398 | 3.71E-10 | 0.01205 | FALSE | 0.002744 | 39.25394 |
| 31 | rs4661543 | 0.274437 | 0.042376 | 9.40E-11 | 0.1188 | FALSE | 0.002931 | 41.93675 |
| 32 | rs4916215 | 0.223144 | 0.033969 | 5.07E-11 | 0.9655 | FALSE | 0.003015 | 43.14558 |
| 33 | rs58688157 | -0.22314 | 0.033565 | 2.97E-11 | 0.2734 | FALSE | 0.003088 | 44.19204 |
| 34 | rs58721818 | 0.65752 | 0.075594 | 3.38E-18 | 0.3951 | FALSE | 0.005275 | 75.64518 |
| 35 | rs597808 | -0.16252 | 0.029474 | 3.51E-08 | 0.4806 | FALSE | 0.002127 | 30.40052 |
| 36 | rs6671847 | 0.198851 | 0.028965 | 6.64E-12 | 0.4868 | FALSE | 0.003293 | 47.12427 |
| 37 | rs6679677 | 0.336472 | 0.046485 | 4.55E-13 | 0.005784 | FALSE | 0.003659 | 52.38462 |
| 38 | rs6889239 | 0.277632 | 0.03174 | 2.19E-18 | 0.08806 | FALSE | 0.005334 | 76.50051 |
| 39 | rs7097397 | -0.18633 | 0.028712 | 8.60E-11 | 0.2155 | FALSE | 0.002943 | 42.10986 |
| 40 | rs73050535 | -0.71335 | 0.124134 | 9.11E-09 | 0.1575 | FALSE | 0.002309 | 33.01893 |
| 41 | rs73068668 | -0.31471 | 0.05749 | 4.40E-08 | 0.3325 | FALSE | 0.002096 | 29.96221 |
| 42 | rs7768653 | -0.20701 | 0.029689 | 3.11E-12 | 0.9594 | FALSE | 0.003396 | 48.61211 |
| 43 | rs7823055 | -0.35066 | 0.028621 | 1.64E-34 | 0.7561 | FALSE | 0.010412 | 150.0861 |
| 44 | rs7899626 | 0.182322 | 0.033253 | 4.19E-08 | 0.5622 | FALSE | 0.002103 | 30.05733 |
| 45 | rs9852014 | 0.620577 | 0.049273 | 2.26E-36 | 0.2575 | FALSE | 0.010996 | 158.6053 |

**Supplementary Table 4:** The instrumental variables used in MR analysis between exposure (ulcerative colitis) and outcome (urticaria).

|  | **SNP** | **beta.exposure** | **se.exposure** | **pval.exposure** | **pval.outcome** | **palindromic** | **R2** | **F** |
| --- | --- | --- | --- | --- | --- | --- | --- | --- |
| 1 | rs10090868 | 0.378436 | 0.046109 | 2.26E-16 | 0.9588 | FALSE | 0.002545 | 67.35637 |
| 2 | rs10148332 | -0.43826 | 0.050796 | 6.26E-18 | 0.6627 | FALSE | 0.002811 | 74.43149 |
| 3 | rs10448585 | 0.039221 | 0.004785 | 2.46E-16 | 0.9514 | FALSE | 0.002538 | 67.18945 |
| 4 | rs10499206 | -0.74194 | 0.054402 | 2.38E-42 | 0.2286 | FALSE | 0.006995 | 185.9809 |
| 5 | rs10499312 | -0.59884 | 0.05247 | 3.60E-30 | 0.6382 | FALSE | 0.004909 | 130.2469 |
| 6 | rs10514388 | -0.55389 | 0.074158 | 8.08E-14 | 0.6964 | FALSE | 0.002108 | 55.78181 |
| 7 | rs10758669 | 0.14842 | 0.020751 | 8.52E-13 | 0.1766 | FALSE | 0.001934 | 51.15481 |
| 8 | rs10781977 | 0.139762 | 0.023901 | 4.99E-09 | 0.3672 | FALSE | 0.001293 | 34.19082 |
| 9 | rs10799837 | 0.10436 | 0.018131 | 8.62E-09 | 0.2252 | FALSE | 0.001253 | 33.12737 |
| 10 | rs10906102 | 0.476234 | 0.048668 | 1.30E-22 | 0.07239 | FALSE | 0.003613 | 95.74803 |
| 11 | rs11115323 | -0.39204 | 0.045694 | 9.51E-18 | 0.4712 | FALSE | 0.00278 | 73.60612 |
| 12 | rs11145750 | -0.12222 | 0.019169 | 1.82E-10 | 0.2013 | FALSE | 0.001537 | 40.64794 |
| 13 | rs11209026 | -0.55389 | 0.050474 | 5.12E-28 | 0.8645 | FALSE | 0.00454 | 120.4104 |
| 14 | rs11679814 | -0.47 | 0.080687 | 5.71E-09 | 0.674599 | FALSE | 0.001283 | 33.92854 |
| 15 | rs11716652 | -0.27003 | 0.033904 | 1.66E-15 | 0.9428 | FALSE | 0.002397 | 63.42705 |
| 16 | rs11739663 | -0.13976 | 0.025166 | 2.80E-08 | 0.6507 | FALSE | 0.001167 | 30.8392 |
| 17 | rs11740487 | -0.62594 | 0.053555 | 1.47E-31 | 0.5059 | FALSE | 0.005147 | 136.5959 |
| 18 | rs11885200 | -0.55389 | 0.06115 | 1.33E-19 | 0.8118 | FALSE | 0.003098 | 82.03903 |
| 19 | rs12200541 | -0.43826 | 0.042825 | 1.40E-24 | 0.8781 | FALSE | 0.003951 | 104.7216 |
| 20 | rs12303913 | -0.52473 | 0.043691 | 3.15E-33 | 0.8858 | FALSE | 0.005433 | 144.2286 |
| 21 | rs12565572 | -0.55962 | 0.097055 | 8.12E-09 | 0.9618 | TRUE | 0.001258 | 33.24366 |
| 22 | rs1385133 | -0.28518 | 0.049211 | 6.83E-09 | 0.9014 | FALSE | 0.00127 | 33.58005 |
| 23 | rs1518070 | -0.11333 | 0.020159 | 1.89E-08 | 0.2755 | FALSE | 0.001195 | 31.60207 |
| 24 | rs1543247 | -0.38526 | 0.05157 | 7.98E-14 | 0.94 | TRUE | 0.002109 | 55.80634 |
| 25 | rs1558206 | -0.35066 | 0.062821 | 2.38E-08 | 0.9339 | FALSE | 0.001179 | 31.15452 |
| 26 | rs16905158 | -0.66783 | 0.071653 | 1.16E-20 | 0.1739 | TRUE | 0.003279 | 86.86157 |
| 27 | rs16910532 | -0.45108 | 0.057613 | 4.90E-15 | 0.649901 | FALSE | 0.002316 | 61.29571 |
| 28 | rs16920014 | -0.35767 | 0.049278 | 3.92E-13 | 0.258 | FALSE | 0.001991 | 52.67878 |
| 29 | rs16940202 | -0.16551 | 0.02332 | 1.27E-12 | 0.6984 | FALSE | 0.001904 | 50.37087 |
| 30 | rs16945863 | -0.43826 | 0.047375 | 2.23E-20 | 0.07101 | FALSE | 0.00323 | 85.56909 |
| 31 | rs16948926 | -0.61519 | 0.054955 | 4.35E-29 | 0.9292 | FALSE | 0.004723 | 125.3024 |
| 32 | rs17016573 | -0.37156 | 0.062875 | 3.43E-09 | 0.9365 | FALSE | 0.001321 | 34.92044 |
| 33 | rs17038868 | -0.56531 | 0.102122 | 3.10E-08 | 0.4825 | FALSE | 0.001159 | 30.64135 |
| 34 | rs17066875 | -0.3293 | 0.043401 | 3.26E-14 | 0.9319 | TRUE | 0.002176 | 57.56674 |
| 35 | rs17085007 | -0.14842 | 0.025088 | 3.30E-09 | 0.2978 | FALSE | 0.001324 | 34.99556 |
| 36 | rs17103762 | -0.40547 | 0.06762 | 2.02E-09 | 0.516699 | TRUE | 0.00136 | 35.95161 |
| 37 | rs17135576 | -0.57661 | 0.057613 | 1.40E-23 | 0.094061 | FALSE | 0.003779 | 100.1605 |
| 38 | rs17141433 | -0.37844 | 0.067012 | 1.63E-08 | 0.781601 | TRUE | 0.001206 | 31.88934 |
| 39 | rs17152619 | -0.30011 | 0.048111 | 4.44E-10 | 0.4049 | TRUE | 0.001471 | 38.90625 |
| 40 | rs17337565 | -0.28518 | 0.044943 | 2.22E-10 | 0.8106 | FALSE | 0.001522 | 40.25968 |
| 41 | rs1801274 | -0.19062 | 0.020598 | 2.16E-20 | 0.5833 | FALSE | 0.003233 | 85.63219 |
| 42 | rs2132868 | -0.35767 | 0.060573 | 3.53E-09 | 0.6959 | FALSE | 0.001319 | 34.8644 |
| 43 | rs2233287 | -0.18232 | 0.033017 | 3.35E-08 | 0.8084 | FALSE | 0.001154 | 30.49125 |
| 44 | rs2249237 | -0.73716 | 0.087396 | 3.32E-17 | 0.9754 | TRUE | 0.002687 | 71.13908 |
| 45 | rs2267029 | 0.19062 | 0.032844 | 6.48E-09 | 0.9298 | FALSE | 0.001274 | 33.68236 |
| 46 | rs2283044 | -0.13103 | 0.021038 | 4.72E-10 | 0.1954 | FALSE | 0.001467 | 38.78665 |
| 47 | rs2289057 | -0.35767 | 0.058205 | 7.99E-10 | 0.385 | FALSE | 0.001428 | 37.75959 |
| 48 | rs2291619 | 0.182322 | 0.028782 | 2.38E-10 | 0.4288 | FALSE | 0.001517 | 40.12392 |
| 49 | rs2294711 | -0.30749 | 0.043551 | 1.66E-12 | 0.6173 | FALSE | 0.001884 | 49.84581 |
| 50 | rs2381990 | 0.41871 | 0.048091 | 3.13E-18 | 0.2743 | FALSE | 0.002863 | 75.79978 |
| 51 | rs271461 | -0.53649 | 0.041698 | 6.97E-38 | 0.3989 | TRUE | 0.00623 | 165.5278 |
| 52 | rs2836878 | -0.22314 | 0.022889 | 1.86E-22 | 0.5405 | FALSE | 0.003587 | 95.03922 |
| 53 | rs28456011 | -0.0198 | 0.002469 | 1.05E-15 | 0.2618 | FALSE | 0.002431 | 64.32919 |
| 54 | rs28527282 | -0.04879 | 0.004176 | 1.55E-31 | 0.9134 | FALSE | 0.005143 | 136.4913 |
| 55 | rs2872507 | -0.13976 | 0.021311 | 5.44E-11 | 0.2399 | FALSE | 0.001626 | 43.00887 |
| 56 | rs3024505 | -0.22314 | 0.02666 | 5.76E-17 | 0.4192 | FALSE | 0.002646 | 70.05252 |
| 57 | rs36072737 | -0.03922 | 0.005026 | 6.01E-15 | 0.790901 | FALSE | 0.002301 | 60.89362 |
| 58 | rs3924019 | 0.00995 | 0.001261 | 3.06E-15 | 0.7779 | FALSE | 0.002351 | 62.22289 |
| 59 | rs420737 | -0.42527 | 0.076923 | 3.23E-08 | 0.1879 | FALSE | 0.001156 | 30.56188 |
| 60 | rs4246905 | 0.122218 | 0.022386 | 4.77E-08 | 0.6698 | FALSE | 0.001128 | 29.80601 |
| 61 | rs4262668 | 0.512824 | 0.089764 | 1.11E-08 | 0.636 | FALSE | 0.001235 | 32.63597 |
| 62 | rs4408683 | 0.182322 | 0.030369 | 1.93E-09 | 0.8303 | FALSE | 0.001363 | 36.04067 |
| 63 | rs441641 | -0.43178 | 0.075517 | 1.08E-08 | 0.2371 | FALSE | 0.001237 | 32.68912 |
| 64 | rs4510766 | -0.18232 | 0.022175 | 2.00E-16 | 0.9198 | FALSE | 0.002554 | 67.59797 |
| 65 | rs4676406 | 0.131028 | 0.020175 | 8.32E-11 | 0.7083 | FALSE | 0.001595 | 42.17753 |
| 66 | rs4703635 | -0.41211 | 0.070061 | 4.05E-09 | 0.3656 | FALSE | 0.001309 | 34.59699 |
| 67 | rs4785333 | -0.35767 | 0.043896 | 3.69E-16 | 0.375 | FALSE | 0.002508 | 66.38987 |
| 68 | rs4801320 | -0.43826 | 0.072926 | 1.86E-09 | 0.057659 | FALSE | 0.001366 | 36.11238 |
| 69 | rs6008068 | -0.56531 | 0.061687 | 4.99E-20 | 0.7807 | TRUE | 0.00317 | 83.97679 |
| 70 | rs6017342 | 0.182322 | 0.019548 | 1.09E-20 | 0.2854 | FALSE | 0.003284 | 86.98513 |
| 71 | rs6029933 | -0.86289 | 0.096574 | 4.07E-19 | 0.435 | TRUE | 0.003014 | 79.82908 |
| 72 | rs6114404 | -0.37156 | 0.051475 | 5.26E-13 | 0.4993 | FALSE | 0.001969 | 52.10148 |
| 73 | rs6114452 | -0.41211 | 0.069068 | 2.42E-09 | 0.5419 | TRUE | 0.001347 | 35.59967 |
| 74 | rs621526 | 0.631272 | 0.071907 | 1.65E-18 | 0.2244 | FALSE | 0.00291 | 77.06426 |
| 75 | rs6426833 | 0.262364 | 0.021214 | 3.93E-35 | 0.07078 | FALSE | 0.005759 | 152.9375 |
| 76 | rs6538407 | -0.53063 | 0.049214 | 4.18E-27 | 0.3727 | FALSE | 0.004383 | 116.2458 |
| 77 | rs6584283 | 0.19062 | 0.020379 | 8.46E-21 | 0.5319 | FALSE | 0.003303 | 87.48595 |
| 78 | rs6737520 | -0.47623 | 0.062066 | 1.68E-14 | 0.1797 | FALSE | 0.002225 | 58.87067 |
| 79 | rs6811401 | -0.55962 | 0.073432 | 2.52E-14 | 0.3137 | FALSE | 0.002195 | 58.07316 |
| 80 | rs6894569 | -0.57098 | 0.057405 | 2.61E-23 | 0.1022 | FALSE | 0.003733 | 98.92715 |
| 81 | rs6938876 | -0.37156 | 0.064011 | 6.45E-09 | 0.8756 | FALSE | 0.001274 | 33.69156 |
| 82 | rs7073851 | -0.37844 | 0.043259 | 2.17E-18 | 0.166 | FALSE | 0.00289 | 76.52318 |
| 83 | rs7107438 | -0.3293 | 0.052645 | 3.97E-10 | 0.1353 | FALSE | 0.00148 | 39.12462 |
| 84 | rs7134599 | -0.17395 | 0.020964 | 1.06E-16 | 0.2431 | FALSE | 0.002601 | 68.84925 |
| 85 | rs7343134 | -0.57098 | 0.070238 | 4.32E-16 | 0.2816 | FALSE | 0.002496 | 66.07955 |
| 86 | rs7554511 | -0.17395 | 0.02368 | 2.04E-13 | 0.631499 | FALSE | 0.00204 | 53.96173 |
| 87 | rs7588677 | -0.27003 | 0.044411 | 1.20E-09 | 0.4456 | FALSE | 0.001398 | 36.96649 |
| 88 | rs7608910 | -0.17395 | 0.022675 | 1.70E-14 | 0.6738 | FALSE | 0.002224 | 58.84707 |
| 89 | rs7699253 | -0.62058 | 0.085006 | 2.87E-13 | 0.2868 | FALSE | 0.002014 | 53.29129 |
| 90 | rs798502 | -0.13103 | 0.022994 | 1.21E-08 | 0.723501 | FALSE | 0.001228 | 32.46824 |
| 91 | rs8181924 | -0.42527 | 0.0536 | 2.12E-15 | 0.1376 | FALSE | 0.002378 | 62.94548 |
| 92 | rs907611 | -0.12222 | 0.021927 | 2.49E-08 | 0.2226 | FALSE | 0.001175 | 31.06696 |
| 93 | rs9268853 | -0.33647 | 0.021422 | 1.35E-55 | 0.01319 | FALSE | 0.009257 | 246.6978 |
| 94 | rs9341227 | -0.35767 | 0.050075 | 9.15E-13 | 0.8755 | FALSE | 0.001928 | 51.01442 |
| 95 | rs9366752 | -0.157 | 0.02261 | 3.81E-12 | 0.05849 | FALSE | 0.001823 | 48.21644 |
| 96 | rs9377315 | -0.55962 | 0.041511 | 2.02E-41 | 0.793299 | TRUE | 0.006836 | 181.7268 |
| 97 | rs949681 | -0.39878 | 0.061211 | 7.28E-11 | 0.02324 | FALSE | 0.001605 | 42.43896 |
| 98 | rs9663811 | -0.35767 | 0.055892 | 1.56E-10 | 0.6857 | FALSE | 0.001549 | 40.94893 |
| 99 | rs9822268 | -0.19062 | 0.022374 | 1.60E-17 | 0.4965 | FALSE | 0.002741 | 72.57932 |
| 100 | rs9830968 | -0.46373 | 0.080506 | 8.40E-09 | 0.9414 | FALSE | 0.001255 | 33.1777 |
| 101 | rs984123 | -0.54232 | 0.057173 | 2.41E-21 | 0.1128 | FALSE | 0.003396 | 89.96979 |
| 102 | rs9928368 | -0.43178 | 0.069449 | 5.06E-10 | 0.9606 | FALSE | 0.001462 | 38.65097 |
| 103 | rs9983355 | -0.41871 | 0.068588 | 1.03E-09 | 0.783401 | FALSE | 0.001409 | 37.26436 |

**Supplementary Table 5:** The instrumental variables used in MR analysis between exposure (Crohn's disease) and outcome (urticaria).

|  | **SNP** | **beta.exposure** | **se.exposure** | **pval.exposure** | **pval.outcome** | **palindromic** | **R2** | **F** |
| --- | --- | --- | --- | --- | --- | --- | --- | --- |
| 1 | rs1056441 | 0.167 | 0.0255 | 5.44E-11 | 0.384 | FALSE | 0.00205 | 42.88555 |
| 2 | rs10761659 | 0.212006 | 0.0237 | 3.42E-19 | 0.1008 | FALSE | 0.003817 | 80.01253 |
| 3 | rs11209026 | -0.9952 | 0.0639 | 1.05E-54 | 0.8645 | FALSE | 0.011482 | 242.5362 |
| 4 | rs11236797 | 0.181104 | 0.0231 | 4.85E-15 | 0.1054 | FALSE | 0.002935 | 61.45971 |
| 5 | rs112401990 | 0.132203 | 0.0237 | 2.35E-08 | 0.6738 | FALSE | 0.001488 | 31.11318 |
| 6 | rs114607072 | 0.441797 | 0.0629 | 2.20E-12 | 0.07442 | FALSE | 0.002357 | 49.32904 |
| 7 | rs11564236 | 0.519093 | 0.0595 | 2.85E-18 | 0.8744 | FALSE | 0.003631 | 76.10529 |
| 8 | rs12194825 | -0.1719 | 0.0298 | 8.00E-09 | 0.8991 | FALSE | 0.001591 | 33.27345 |
| 9 | rs1250573 | -0.1709 | 0.0264 | 9.01E-11 | 0.8622 | FALSE | 0.002003 | 41.89954 |
| 10 | rs12692254 | 0.301402 | 0.0232 | 1.86E-38 | 0.2758 | TRUE | 0.008017 | 168.762 |
| 11 | rs12717899 | 0.159198 | 0.0289 | 3.59E-08 | 0.664401 | FALSE | 0.001451 | 30.34156 |
| 12 | rs1297271 | -0.1549 | 0.0237 | 6.28E-11 | 0.4286 | FALSE | 0.002041 | 42.71399 |
| 13 | rs13135092 | 0.221495 | 0.0389 | 1.21E-08 | 0.9861 | FALSE | 0.00155 | 32.41806 |
| 14 | rs1332099 | -0.2116 | 0.0231 | 4.36E-20 | 0.2653 | FALSE | 0.004002 | 83.89836 |
| 15 | rs140054334 | 0.349797 | 0.0628 | 2.57E-08 | 0.500299 | FALSE | 0.001483 | 31.02211 |
| 16 | rs1456896 | 0.139301 | 0.0251 | 2.90E-08 | 0.693999 | FALSE | 0.001473 | 30.79778 |
| 17 | rs147018773 | 0.321699 | 0.0375 | 8.89E-18 | 0.06111 | FALSE | 0.003512 | 73.58602 |
| 18 | rs147684209 | 0.154901 | 0.0244 | 2.34E-10 | 0.02719 | FALSE | 0.001926 | 40.29834 |
| 19 | rs148844907 | 0.958001 | 0.1419 | 1.47E-11 | 0.7899 | FALSE | 0.002178 | 45.57488 |
| 20 | rs151314883 | -0.22399 | 0.0327 | 7.12E-12 | 0.04933 | FALSE | 0.002242 | 46.91759 |
| 21 | rs1873625 | 0.180704 | 0.0243 | 1.09E-13 | 0.3627 | FALSE | 0.002641 | 55.29443 |
| 22 | rs1887428 | -0.1681 | 0.0243 | 4.22E-12 | 0.09436 | FALSE | 0.002286 | 47.84936 |
| 23 | rs1932990 | 0.152901 | 0.0263 | 6.02E-09 | 0.233 | FALSE | 0.001616 | 33.79618 |
| 24 | rs2076756 | 0.399806 | 0.0242 | 3.24E-61 | 0.2742 | FALSE | 0.012901 | 272.9143 |
| 25 | rs2129944 | -0.1562 | 0.0271 | 7.81E-09 | 0.8202 | FALSE | 0.001588 | 33.21864 |
| 26 | rs2188962 | 0.212398 | 0.0228 | 1.36E-20 | 0.1118 | FALSE | 0.004138 | 86.77399 |
| 27 | rs2505640 | -0.1457 | 0.0237 | 7.61E-10 | 0.6851 | FALSE | 0.001807 | 37.79086 |
| 28 | rs281379 | 0.139797 | 0.0238 | 4.26E-09 | 0.5268 | FALSE | 0.001649 | 34.4985 |
| 29 | rs28701841 | 0.224303 | 0.0373 | 1.85E-09 | 0.1259 | FALSE | 0.001729 | 36.15854 |
| 30 | rs3024505 | 0.177903 | 0.0302 | 3.90E-09 | 0.4192 | FALSE | 0.001659 | 34.69853 |
| 31 | rs3091315 | -0.1795 | 0.0263 | 9.52E-12 | 0.782899 | FALSE | 0.002226 | 46.57798 |
| 32 | rs3810936 | 0.207799 | 0.0263 | 2.46E-15 | 0.745901 | FALSE | 0.00298 | 62.42144 |
| 33 | rs4077515 | 0.215901 | 0.0235 | 4.37E-20 | 0.2594 | FALSE | 0.004026 | 84.39797 |
| 34 | rs444210 | 0.163402 | 0.0229 | 1.02E-12 | 0.068461 | FALSE | 0.002432 | 50.90989 |
| 35 | rs4851586 | -0.1689 | 0.0261 | 9.94E-11 | 0.002496 | FALSE | 0.002001 | 41.87276 |
| 36 | rs4902642 | -0.1292 | 0.0236 | 4.34E-08 | 0.8537 | FALSE | 0.001433 | 29.96719 |
| 37 | rs4921497 | 0.160298 | 0.0244 | 5.49E-11 | 0.9471 | FALSE | 0.002062 | 43.15538 |
| 38 | rs56062135 | 0.193097 | 0.0269 | 7.45E-13 | 0.7466 | FALSE | 0.002461 | 51.52345 |
| 39 | rs6588243 | 0.131704 | 0.0234 | 1.78E-08 | 0.6967 | FALSE | 0.001515 | 31.67558 |
| 40 | rs6704109 | 0.202002 | 0.0256 | 2.77E-15 | 0.733701 | FALSE | 0.002973 | 62.25723 |
| 41 | rs6873866 | -0.1681 | 0.0239 | 2.06E-12 | 0.6604 | FALSE | 0.002363 | 49.46265 |
| 42 | rs697693 | 0.172296 | 0.0281 | 8.36E-10 | 0.2508 | FALSE | 0.001797 | 37.59206 |
| 43 | rs72798422 | 0.590392 | 0.0508 | 3.19E-31 | 0.3089 | FALSE | 0.006426 | 135.0554 |
| 44 | rs744166 | -0.1293 | 0.0233 | 2.92E-08 | 0.8619 | FALSE | 0.001472 | 30.79193 |
| 45 | rs7543234 | 0.155498 | 0.0267 | 6.10E-09 | 0.4633 | FALSE | 0.001622 | 33.91451 |
| 46 | rs7713270 | 0.296602 | 0.0241 | 6.97E-35 | 0.3229 | FALSE | 0.007201 | 151.4511 |
| 47 | rs78487399 | 0.2259 | 0.037 | 1.03E-09 | 0.7475 | FALSE | 0.001782 | 37.27241 |
| 48 | rs80262450 | 0.283102 | 0.0353 | 1.08E-15 | 0.9809 | FALSE | 0.00307 | 64.31242 |
| 49 | rs8137950 | 0.173996 | 0.0286 | 1.17E-09 | 0.8197 | FALSE | 0.001769 | 37.00879 |
| 50 | rs8178977 | 0.1928 | 0.0274 | 2.06E-12 | 0.1169 | FALSE | 0.002365 | 49.50754 |
| 51 | rs907092 | 0.130396 | 0.0228 | 1.01E-08 | 0.2181 | FALSE | 0.001564 | 32.70523 |
| 52 | rs921720 | 0.162895 | 0.0237 | 6.40E-12 | 0.003192 | FALSE | 0.002257 | 47.23645 |

**Supplementary Table 6:** The instrumental variables used in MR analysis between exposure (urticaria) and outcome (rheumatoid arthritis).

|  | **SNP** | **beta.exposure** | **se.exposure** | **pval.exposure** | **pval.outcome** | **palindromic** | **R2** | **F** |
| --- | --- | --- | --- | --- | --- | --- | --- | --- |
| 1 | rs10925146 | 0.1075 | 0.0231 | 3.11E-06 | 0.222 | TRUE | 9.95E-05 | 21.65654 |
| 2 | rs11143793 | 0.0941 | 0.0206 | 5.00E-06 | 0.762801 | FALSE | 9.59E-05 | 20.86608 |
| 3 | rs12580889 | 0.2695 | 0.0543 | 6.92E-07 | 0.1147 | FALSE | 0.000113 | 24.63281 |
| 4 | rs12936485 | -0.1556 | 0.032 | 1.12E-06 | 0.9639 | FALSE | 0.000109 | 23.64369 |
| 5 | rs13105680 | 0.2347 | 0.0493 | 1.93E-06 | 0.4366 | FALSE | 0.000104 | 22.66357 |
| 6 | rs167941 | -0.1167 | 0.0238 | 9.80E-07 | 0.4684 | FALSE | 0.000111 | 24.04273 |
| 7 | rs1980496 | 0.1142 | 0.0234 | 1.06E-06 | 3.30E-154 | FALSE | 0.000109 | 23.81752 |
| 8 | rs205002 | -0.1 | 0.0215 | 3.25E-06 | 3.29E-07 | FALSE | 9.94E-05 | 21.63312 |
| 9 | rs4258792 | -0.1083 | 0.0235 | 3.98E-06 | 0.8837 | FALSE | 9.76E-05 | 21.23818 |
| 10 | rs4844846 | 0.1449 | 0.0269 | 6.91E-08 | 0.3693 | FALSE | 0.000133 | 29.01538 |
| 11 | rs56043070 | 0.2429 | 0.0455 | 9.28E-08 | 0.323 | FALSE | 0.000131 | 28.49891 |
| 12 | rs6035202 | -0.0951 | 0.0207 | 4.16E-06 | 0.1569 | FALSE | 9.70E-05 | 21.10651 |
| 13 | rs652197 | -0.1201 | 0.0258 | 3.13E-06 | 0.6294 | FALSE | 9.96E-05 | 21.66919 |
| 14 | rs6787175 | -0.1016 | 0.0204 | 6.75E-07 | 0.02078 | TRUE | 0.000114 | 24.80408 |
| 15 | rs6901866 | 0.1037 | 0.0226 | 4.43E-06 | 0.322 | FALSE | 9.68E-05 | 21.0541 |
| 16 | rs74801096 | 0.3232 | 0.0702 | 4.11E-06 | 0.791099 | FALSE | 9.74E-05 | 21.19652 |
| 17 | rs9982936 | 0.321699 | 0.0375 | 8.89E-18 | 0.06111 | FALSE | 0.003512 | 73.58602 |

**Supplementary Table 7:** The instrumental variables used in MR analysis between exposure (urticaria) and outcome (systemic lupus erythematosus).

|  | **SNP** | **beta.exposure** | **se.exposure** | **pval.exposure** | **pval.outcome** | **palindromic** | **R2** | **F** |
| --- | --- | --- | --- | --- | --- | --- | --- | --- |
| 1 | rs10925146 | 0.1075 | 0.0231 | 3.11E-06 | 0.075232 | TRUE | 9.95E-05 | 21.65654 |
| 2 | rs11143793 | 0.0941 | 0.0206 | 5.00E-06 | 0.833756 | FALSE | 9.59E-05 | 20.86608 |
| 3 | rs12580889 | 0.2695 | 0.0543 | 6.92E-07 | 0.458812 | FALSE | 0.000113 | 24.63281 |
| 4 | rs12936485 | -0.1556 | 0.032 | 1.12E-06 | 0.234394 | FALSE | 0.000109 | 23.64369 |
| 5 | rs13105680 | 0.2347 | 0.0493 | 1.93E-06 | 0.945051 | FALSE | 0.000104 | 22.66357 |
| 6 | rs167941 | -0.1167 | 0.0238 | 9.80E-07 | 0.076628 | TRUE | 0.000111 | 24.04273 |
| 7 | rs1980496 | 0.1142 | 0.0234 | 1.06E-06 | 1.01E-15 | FALSE | 0.000109 | 23.81752 |
| 8 | rs205002 | -0.1 | 0.0215 | 3.25E-06 | 4.39E-17 | FALSE | 9.94E-05 | 21.63312 |
| 9 | rs4258792 | -0.1083 | 0.0235 | 3.98E-06 | 0.01776 | FALSE | 9.76E-05 | 21.23818 |
| 10 | rs4844846 | 0.1449 | 0.0269 | 6.91E-08 | 0.850656 | FALSE | 0.000133 | 29.01538 |
| 11 | rs56043070 | 0.2429 | 0.0455 | 9.28E-08 | 0.045154 | FALSE | 0.000131 | 28.49891 |
| 12 | rs652197 | -0.1201 | 0.0258 | 3.13E-06 | 0.580314 | FALSE | 9.96E-05 | 21.66919 |
| 13 | rs6787175 | -0.1016 | 0.0204 | 6.75E-07 | 0.391417 | TRUE | 0.000114 | 24.80408 |
| 14 | rs6901866 | 0.1037 | 0.0226 | 4.43E-06 | 0.053151 | FALSE | 9.68E-05 | 21.0541 |
| 15 | rs74801096 | 0.3232 | 0.0702 | 4.11E-06 | 0.226023 | TRUE | 9.74E-05 | 21.19652 |
| 16 | rs9982936 | 0.1367 | 0.0287 | 1.90E-06 | 0.398686 | FALSE | 0.000104 | 22.68659 |

**Supplementary Table 8:** The instrumental variables used in MR analysis between exposure (urticaria) and outcome (ulcerative colitis).

|  | **SNP** | **beta.exposure** | **se.exposure** | **pval.exposure** | **pval.outcome** | **palindromic** | **R2** | **F** |
| --- | --- | --- | --- | --- | --- | --- | --- | --- |
| 1 | rs10925146 | 0.1075 | 0.0231 | 3.11E-06 | 0.763999 | TRUE | 9.95E-05 | 21.65654 |
| 2 | rs11143793 | 0.0941 | 0.0206 | 5.00E-06 | 0.719999 | FALSE | 9.59E-05 | 20.86608 |
| 3 | rs12936485 | -0.1556 | 0.032 | 1.12E-06 | 0.66 | FALSE | 0.000109 | 23.64369 |
| 4 | rs4258792 | -0.1083 | 0.0235 | 3.98E-06 | 0.778999 | FALSE | 9.76E-05 | 21.23818 |
| 5 | rs6035202 | -0.0951 | 0.0207 | 4.16E-06 | 0.411 | FALSE | 9.70E-05 | 21.10651 |
| 6 | rs6901866 | 0.1037 | 0.0226 | 4.43E-06 | 0.139 | FALSE | 9.68E-05 | 21.0541 |
| 7 | rs9982936 | 0.1367 | 0.0287 | 1.90E-06 | 0.169 | FALSE | 0.000104 | 22.68659 |

**Supplementary Table 9:** The instrumental variables used in MR analysis between exposure (urticaria) and outcome (Crohn's disease).

|  | **SNP** | **beta.exposure** | **se.exposure** | **pval.exposure** | **pval.outcome** | **palindromic** | **R2** | **F** |
| --- | --- | --- | --- | --- | --- | --- | --- | --- |
| 1 | rs10925146 | 0.1075 | 0.0231 | 3.11E-06 | 0.878 | FALSE | 9.95E-05 | 21.65654 |
| 2 | rs11143793 | 0.0941 | 0.0206 | 5.00E-06 | 0.9165 | FALSE | 9.59E-05 | 20.86608 |
| 3 | rs12580889 | 0.2695 | 0.0543 | 6.92E-07 | 0.2496 | FALSE | 0.000113 | 24.63281 |
| 4 | rs12936485 | -0.1556 | 0.032 | 1.12E-06 | 0.8587 | FALSE | 0.000109 | 23.64369 |
| 5 | rs13105680 | 0.2347 | 0.0493 | 1.93E-06 | 0.06586 | FALSE | 0.000104 | 22.66357 |
| 6 | rs167941 | -0.1167 | 0.0238 | 9.80E-07 | 0.1733 | FALSE | 0.000111 | 24.04273 |
| 7 | rs4258792 | -0.1083 | 0.0235 | 3.98E-06 | 0.8305 | FALSE | 9.76E-05 | 21.23818 |
| 8 | rs4844846 | 0.1449 | 0.0269 | 6.91E-08 | 0.6556 | FALSE | 0.000133 | 29.01538 |
| 9 | rs56043070 | 0.2429 | 0.0455 | 9.28E-08 | 0.087531 | FALSE | 0.000131 | 28.49891 |
| 10 | rs6035202 | -0.0951 | 0.0207 | 4.16E-06 | 0.645199 | FALSE | 9.70E-05 | 21.10651 |
| 11 | rs652197 | -0.1201 | 0.0258 | 3.13E-06 | 0.03269 | FALSE | 9.96E-05 | 21.66919 |
| 12 | rs6787175 | -0.1016 | 0.0204 | 6.75E-07 | 0.1175 | TRUE | 0.000114 | 24.80408 |
| 13 | rs6901866 | 0.1037 | 0.0226 | 4.43E-06 | 0.2874 | FALSE | 9.68E-05 | 21.0541 |
| 14 | rs74801096 | 0.3232 | 0.0702 | 4.11E-06 | 0.619599 | FALSE | 9.74E-05 | 21.19652 |
| 15 | rs76036679 | 0.6216 | 0.1358 | 4.68E-06 | 0.9348 | FALSE | 9.63E-05 | 20.95166 |
| 16 | rs76392654 | 0.2087 | 0.0416 | 5.41E-07 | 0.3876 | FALSE | 0.000116 | 25.16832 |
| 17 | rs9982936 | 0.1367 | 0.0287 | 1.90E-06 | 0.001449 | FALSE | 0.000104 | 22.68659 |

**Supplementary Table 10:** MR analysis process of exposures and outcomes.

| **Exposure** | **Outcome** | **Random-effects IVW** | | **Heterogeneity** | | **Pleiotropy** | **MR-PRESSO** | | | **MR-RAPS** |
| --- | --- | --- | --- | --- | --- | --- | --- | --- | --- | --- |
|  |  | **P value** | **OR (95%CI)** | **Cochran’s Q Test (IVW)** | **Rucker’s** **Q Test (MR-Egger)** | **Intercept Test**  **(MR-Egger)** | **Outliers**  **Test** | | **Pleiotropy Test** | **Normal**  **Distribution** |
|  |  |  |  | **P value** | **P value** | **P value** | **Significant** | **Potential** | **P value** | **P value** |
| RA-1 | Urticaria | < 0.001 | 1.067 (1.028 - 1.107) | 0.011 | 0.010 | 0.470 | 1 | 3 | 0.003 | 0.700 |
| SLE-1 | Urticaria | 0.106 | 1.024 (0.995 - 1.054) | 0.030 | 0.033 | 0.287 | 0 | 3 | 0.031 | 0.997 |
| Urticaria | SLE-1 | 0.303 | 1.152 (0.880 - 1.509) | 0.099 | 0.133 | 0.261 | 1 | 0 | 0.046 | 0.925 |
| Urticaria | CD-1 | 0.615 | 0.960 (0.820 - 1.125) | 0.034 | 0.024 | 0.855 | 1 | 2 | 0.027 | 0.918 |

MR: mendelian randomization; IVW: inverse variance weighted; RA: rheumatoid arthritis; SLE: systemic lupus erythematosus; UC: ulcerative colitis; CD: Crohn's disease.
